# Maintaining genetic stability in sweet potato: epigenetic insights into propagation and drought tolerance

**DOI:** 10.3389/fpls.2026.1807723

**Published:** 2026-05-05

**Authors:** Ajay Dhungana, Imana L. Power, Prasad Gandham, Niranjan Baisakh, Jeffrey C. Gregorie, Catherine DeRobertis, Don R. La Bonte

**Affiliations:** 1 School of Plant, Environmental, and Soil Sciences, Louisiana State University AgCenter, Baton Rouge, LA, United States; 2 Department of Plant Pathology and Crop Physiology, Louisiana State University AgCenter, Baton Rouge, LA, United States

**Keywords:** bisulfite sequencing, drought, methylation, sweetpotato, tissue culture

## Abstract

Sweetpotato is propagated vegetatively via traditional vine cutting or tissue culture using node or meristematic tissues, yet phenotypic variations among the plants are observed irrespective of the propagation method due to environmental interactions leading to mutations and/or epigenetic modifications. Methylation-mediated epigenetic modification refers to the addition of methyl groups to DNA, primarily at cytosine bases, which regulates gene expression without altering the DNA sequence. The present study focused on understanding how various propagation methods and environmental stressors (drought) affect DNA methylation patterns and epigenetic stability in commercial varieties and half-sib progenies using bisulfite sequencing. This study revealed genotype-specific DNA methylation responses to tissue culture and drought stress in sweetpotato, reflecting genotype-dependent epigenetic plasticity. Statistical analyses revealed that methylation patterns remained largely stable during tissue culture, with only minor, genotype-specific fluctuations across Meristem initiated and conventional nodal cultures. In contrast, drought stress induced unpredictable, variety-dependent methylation remodeling, reflecting active epigenetic reprogramming. Prolonged tissue culture resulted in DNA methylation enhancement, most likely due to cumulative *in vitro* stress; varieties maintained as greenhouse mother plants, displayed pronounced drought-induced methylation shifts. The consistent stability observed in clonal *in vitro* subculture supports the feasibility of long-term *in vitro* propagation for maintaining epigenetic stability.

## 1 Introduction

Sweetpotato [*Ipomoea batatas* (L.) Lam.] is traditionally propagated vegetatively through vine cuttings or sprouts from storage roots to maintain clonal uniformity (Gaba and Singer, 2009). Modern approaches, such as tissue culture, are now widely used for clonal propagation, genetic improvement, and the production of disease-free and stress-resilient plants (Hussain et al., 2012; Sello et al., 2018). However, phenotypic variation among plants persists due to both genetic (spontaneous mutations) and environmental interactions. Propagation via nodal culture using preformed meristematic tissues significantly reduces genomic variation and ensures greater genetic stability (Villordon and LaBonte, 1996; Quiroz et al., 2017). Molecular marker studies have confirmed that node-derived plants exhibit markedly fewer DNA polymorphisms than those propagated adventitiously from storage roots, suggesting that meristematic regions minimize somatic mutations and preserve epigenetic stability (Nguyen and Gutzat, 2022; Villordon and LaBonte, 1996). Despite these benefits, tissue culture poses risks such as somaclonal variation/chimerism (Akomeah et al., 2019).

Research on *in vitro* propagation often reveals abnormal phenotypic changes, known as somaclonal variation, arising from genetic and/or epigenetic alteration in several crop species, including maize (Stelpflug et al., 2014), cotton (Lu et al., 2017) and cocoa (Rodríguez López et al., 2010). Epigenetic changes refer to heritable changes that alter phenotypes without changing the gene coding sequence or promoter region (Rapp and Wendel, 2005). These changes are primarily mediated by DNA methylation, histone modifications, and transposable elements, which regulate gene activity (Hirsch and Springer, 2017; Cara et al., 2020). Among these mechanisms, DNA methylation, defined as the addition of a methyl group (-CH_3_) to the fifth carbon of cytosine, is a major regulator of genomic imprinting, transposon silencing, and cellular differentiation (Rapp and Wendel, 2005). DNA methylation alters genomic stability, affecting gene expression by altering chromatin structure (Lukens and Zhan, 2007). DNA methylation occurs in CG, CHG, and CHH contexts, maintained by DNA methyltransferase (MET1, CMT3, CMT2) in plants (Law and Jacobsen, 2010). As key regulators, cytosine-5 DNA methyltransferases (C5-MTases) and DNA demethylases (dMTases) govern the dynamic patterns of DNA methylation (Yang et al., 2025). The C5-MTases add methyl groups to the DNA while the dMTases remove them, thereby maintaining epigenetic plasticity. These changes can occur spontaneously or can be influenced by genetic and environmental factors. Epigenetic regulation, studied extensively in *Arabidopsis thaliana*, helps plants adapt to unpredictable environments by enabling metastable changes in gene activity (Pikaard and Mittelsten Scheid, 2014). Sweetpotato, with its large and complex genome (Wu et al., 2018), possesses high levels of transposable elements (TEs) and methylated DNA (Akomeah et al., 2019). Next-generation sequencing (NGS) costs are declining, making whole-genome bisulfite sequencing (WGBS) more accessible for fundamental and applied research (Schmitz et al., 2013; Wu et al., 2018; Selby et al., 2025). The WGBS approach has been instrumental in mapping methylomes across various plant species, providing valuable insights into epigenetic regulation (Xiong et al., 1999).

In clonally propagated crops, understanding how genetic and epigenetic changes influence phenotypic traits is essential for ensuring varietal fidelity and crop performance. For instance, sweetpotato varieties such as ‘Beauregard’, released in 1987 (Rolston et al., 1987) and ‘Covington’, released in 2005 (Yencho et al., 2008), are dominant in the southeastern United States due to their superior storage root shape, disease resistance, and adaptability. These varieties have been widely grown and extensively propagated through both traditional and *in vitro* techniques for years, underscoring the need to maintain their original genetic fidelity. Beyond these primary varieties, other established varieties like ‘Bonita’ (La Bonte et al., 2011) (white-flesh), ‘Bellevue’ (La Bonte et al., 2015) (orange-flesh), and ‘Murasaki-29’ (La Bonte et al., 2008) (white-flesh), serve as standard checks and vital parental lines in breeding nurseries. Investigating these distinct phenotypes offers a unique opportunity to determine whether epigenetic regulation differs between orange and white-fleshed varieties, particularly regarding stress signaling. ‘Murasaki-29’, where anecdotal evidence from growers indicates decline in yield over successive years of cultivation when growers used virus tested clean materials suggests that accumulated genetic or epigenetic modifications may be compromising its long-term performance and commercial viability. Given their extensive use and long propagation histories, these varieties serve as important examples of sweetpotato that may have accumulated genetic or epigenetic variation over time. Especially considering the lack of knowledge related to the impact of propagation-related and environmental stresses, such as tissue culture and drought on genomic stability and DNA methylation patterns in sweetpotato.

The present study focused on half-sib progeny lines generated from three commercially important varieties, ‘Bellevue’, ‘Bonita’ and ‘Murasaki-29’ to investigate epigenetic variation in response to tissue culture and drought stress on greenhouse-maintained plants of these parental varieties (**Supplementary Figure 1**; **Supplementary Table 1**). These studies represent the first comprehensive analysis of methylation landscapes integrating genotypic responses in both tissue culture-derived progenies and drought-stressed plants in sweetpotato.

## 2 Materials and methods

### 2.1 Sampling and plant growth

True seeds from three sweet potato varieties, ‘Murasaki-29’, ‘Bellevue’, and ‘Bonita’, were randomly selected in January 2023. These half-sib progenies were sown in soilless growing media (Sungro; Sun Gro Horticulture, Bellevue, WA) at a depth of 2–3 cm in a greenhouse at the LSU AgCenter (Louisiana State University Agricultural Center), Sweet Potato Research Station, Chase, LA (32°6’N, 91°42’W). The greenhouse was maintained at an average temperature of 27.9 °C during the day and 23.4 °C at night, with plants grown under natural light (**Supplementary Table 2**). Water was provided for 3 min 3 times per day, representing optimal production conditions. Three half-sib progenies from each variety were selected at random and subdivided for clonal maintenance once plants reached approximately 20 cm in height. Two tissue culture methods were tested: Meristem initiated nodal culture and conventional nodal culture (**Supplementary Figure 2**). A greenhouse mother plant of each half-sib was used for comparison.

### 2.2 Meristem initiated nodal culture

Meristem tip culture is a common method for generating virus-free plantlets, as meristematic regions lack vascular tissue and have high cell division activity (Wondimu et al., 2012). Meristematic apical tip tissues were artificially cultured in Murashige and Skoog (MS) basal medium (Murashige and Skoog, 1962) (Phytotechnology Laboratories) supplemented with vitamins, sucrose (30 g/L), gellan gum (2.5 g/L), and 0.5 mg/L 6-benzylaminopurine. The medium was adjusted to a final volume of 1 L with distilled water at a pH 5.25–6.25 and autoclaved at 121°C for 20 min. The meristematic tips required approximately four months to regenerate into complete plantlets with well-developed shoots and roots. These plantlets were subsequently propagated via five rounds of subculturing using nodal cuttings in a hormone-free MS medium. The first four subcultures were performed at ~4-week intervals, while the final subculture was extended to a 6-week interval. Leaf samples were collected at each subculture stage and stored frozen at -80°C for DNA extraction. This study served as the initial explant source for evaluating the impact of meristematic apical tip-initiated regeneration on (epi)genetic stability.

### 2.3 Conventional nodal culture

Nodal segments collected from the mid-section of a healthy grown half-sib sweetpotato plant were used as explants. Surface sterilization was performed by rinsing in 70% ethanol for 1 min, followed by immersion in 10% commercial bleach solution (0.625% NaOCl) containing 50 µl/L Tween-20 for 10 min, with occasional swirling. Explants were subsequently rinsed three times with sterile distilled water under aseptic conditions. Sterilized nodal segments were cultured on MS medium (pH 5.6–5.8), solidified with gellan gum and supplemented with 30 g/L sucrose. These nodes were sub-cultured five times. The first four subcultures were conducted at approximately 4-week intervals, while the final subculture was extended to 6 weeks. A leaf from the first nodal cutting (first regenerants) and subsequent regenerants stage until the fifth cutting stage was used for DNA extraction. At the start of the experiment, DNA was extracted from these mother plants and used as a baseline control for methylation comparisons in all downstream analyses. To ensure equivalence, both meristem-derived and nodal cultures were propagated under identical subculturing timelines.

### 2.4 Drought stress culture

Unlike the tissue culture experiment, which involved half sib progenies, three parental sweetpotato varieties (‘Murasaki-29’, ‘Bellevue’, and ‘Bonita’) were used as the plant material for this study. This experiment was carried out during summer (21 June - 9 August 2023). Plant cuttings (~20 cm length, 4.0 mm diameter) were obtained from the greenhouse at the LSU AgCenter Sweetpotato Research Station, Chase, LA. Washed River sand was used as the growth substrate for all experiments. Unrooted cuttings (slips) were transplanted into approx. 2.2 kg of sand in each plastic pot (10.16 cm width and 24.13 cm height). Each pot had drain holes and detachable plastic bottom plugs. Plants (one plant/pot) were grown in the greenhouse under average temperature of 33.2°C during the day and 25.8°C at night, with artificial lighting provided (**Supplementary Table 2**). Drought stress was imposed by withholding water for 7 days (intermediate drought) and 14 days (drought), while control plants received water throughout the experiment period (Villordon et al., 2018). The drought treatment protocol consisted of several phases. Initially, all plants received 200 ml of water per pot three times a week for the first 20 days after transplanting (DAT). Watering was then stopped for all plants except control after 20 DAT. Following 7 days without water, intermediate drought and control plants received 175 ml of water three times a week, while drought plants received no water for a total of 14 days. After 14 days of drought treatment, these plants were watered again with 175 ml of water three times a week. After 3 days of resumed watering, all plants in the study were cut back to retain 3–5 nodes per plant. New leaves that emerged 13 days post cutback were collected for DNA extraction.

### 2.5 DNA extraction and whole genome bisulfite sequencing

Leaf tissues from 42 various treatments were stored at -80°C for DNA extraction. Three biological replicates per treatment were used for collection of leaf tissues and DNA isolation. Equimolar concentration (15 µg) of DNA from the replicates were pooled prior to sequencing due to limited financial resources, yet capturing all possible methylation incidences across replicates. Approximately 30-40 µg of DNA from each leaf sample was used for whole-genome bisulfite sequencing (WGBS). DNA extraction was performed using the Cetyltrimethylammonium bromide (CTAB) protocol for sequencing (Murray and Thompson, 1980). The extracted DNA was used for bisulfite sequencing in a commercial sequencing facility (Psomagen Inc.; Rockville, MD, USA), using their custom pipeline. Briefly, genomic DNA (gDNA) was quantified using Picogreen assay (Thermo Fisher Scientific; Waltham, MA, USA) on VictorX2 multilabel plate reader (PerkinElmer Inc.; Shelton, CT, USA) and DNA integrity was checked using a gDNA screen tape (Agilent Technologies; Santa Clara, CA, USA).

One hundred nanograms of gDNA was used for whole genome bisulfite library construction with xGen Methyl-Seq DNA prep kit (IDT Inc). The gDNA was fragmented to a size of 350bp followed by bisulfite conversion using the EZ DNA Methylation-Gold Kit (Zymo Research). The bisulfite converted DNA was used as a starting material for library construction. Whole genome bisulfite sequencing library preparation and indexing was carried out using the Methyl-Seq Library Prep protocol. The size-validated final library was normalized to 10 nM and then diluted to 160 pM for loading onto NovaseqX for sequencing. The base call binary files were converted into FASTQ files using bcl2fastq (Illumina Inc.; San Diego, CA, USA).

### 2.6 Post-sequencing bioinformatics analysis

Adapter sequences and low-quality bases were trimmed from raw FASTQ files using Trimmomatic v0.36 (Bolger et al., 2014) with the default parameters using SLIDINGWINDOW:4:15 and MINLEN:50. The resulting high-quality reads were aligned to the *Ipomoea batatas* ‘Beauregard’ genome assembly (version 3) (https://sweetpotato.uga.edu/sweetgains_Beauregard_v3_asm_anno.shtml) using Bismark v0.20.0, with the following parameters: -q --score_min L,0,-0.2 --directional --ignore-quals --no-mixed --no-discordant --dovetail --maxins 500, and --bowtie2. Post-alignment, BAM files were sorted and deduplicated using Bismark tools (Krueger and Andrews, 2011). Methylation calls were extracted using the Bismark methylation extractor, retaining only those cytosine positions covered by a minimum read depth of ≥5 on both DNA strands. These high-confidence sites were used for all downstream analyses. To further classify methylation sites, custom in-house perl scripts were employed. The methylation sites were annotated based on sequence context (CpG, CHG, CHH), genomic region (intergenic, exonic, intronic), and methylation intensity was categorized as hypomethylated (≤30%), intermediately methylated (>30% to <70%), or hypermethylated (≥70%). The number and proportion of methylated cytosines in each sequence context were quantified across all 42 biological samples, providing a comprehensive profile of methylation distribution in the sweetpotato genome.

### 2.7 Statistical analysis

Potential differences in genome-wide and mean DNA methylation levels for both tissue culture (meristem initiated nodal culture, conventional nodal culture and mother plant) and drought stress (control, intermediate drought, and severe drought) conditions for all half-sibs and varieties were assessed using descriptive and statistical test performed separately in R (R Core Team, 2023). In the absence of replicated data, paired wise (non-parametric) tests were considered to evaluate whether median gene-level methylation differs significantly among the tissue culture stages (relative to mother plant) and drought stress (relative to control) within each variety with Benjamini-Hochberg correction applied to control the false discovery rate (FDR). Median differences were used to estimate genome wide effect size and variability across loci were used to highlight substantial intra-genomic variability. A principal component analysis (PCA) was performed to reduce the dimensionality of the dataset and identify underlying methylation patterns among half-sib progenies and varieties. Analysis of variance (ANOVA) was performed to test significant differences in chromosome-wise counts of hypermethylated exonic positions across all methylation contexts (CG, CHG, and CHH) among half-sib progenies and varieties. Post Anova, Fisher’s Least Significant Difference (LSD) were performed at the significance level of *p* < 0.05. Hierarchical clustering of common hypermethylated genes was employed to visualize the high-dimensional methylation landscape, revealing distinct clusters of shared epigenetic patterns across all half-sib progenies and varieties. Subsequently, number of overlapping and commonly methylated genes were generated for both conditions (tissue culture and drought stress) across all varieties (‘Bellevue’, ‘Bonita’ and ‘Murasaki-29’) and half-sib progenies and variety-specific for all drought stress treatments and half-sib progenies specific across all tissue culture stages. The analysis was conducted in R Studio, using base R and the dplyr package for data manipulation. Visualization of these results, such as bar diagram, heatmaps, boxplots, venn-diagram and PCA, was performed in R Studio. Finally, gene annotations of differentially methylated genes (DMGs) were performed through sequence similarity searches against the *Ipomoea batatas* Beauregard (v3) reference genome to infer potential roles in drought (drought vs. control) and tissue culture stress response pathways.

## 3 Results

### 3.1 Whole genome bisulfite sequencing bioinformatics result

Whole-genome bisulfite sequencing (WGBS) was performed to assess genome-wide methylation profiles across propagation methods, drought stress treatments, and varieties. Following quality control, an average of 94.95% of sequence reads across all samples with >Q30 were used in subsequent analysis (**Supplementary Table 3**). Of the 985,099,340 cytosine (C) bases on both strands of the *Ipomoea batatas* ‘Beauregard’ genome,117,065,070 (11.88%) were identified as methylated. Strand-specific analysis revealed a nearly equal distribution of methylated cytosines with 58,555,260 (50.02%) on the plus strand and 58,509,810 (49.98%) on the minus strand. The distribution of raw methylation sites across all reference chromosomes showed that 6487 out of 6629 scaffolds had at least one raw methylation call. To enhance reliability, methylation sites were filtered using a minimum read depth cutoff of ≥5, resulting in 2672 scaffolds with high-confidence methylation calls (**Supplementary Table 4**). A comprehensive summary table detailing the number of methylation calls per chromosome and genes across all 42 samples (**Table 1**).

**Table 1:** The summary statistics of different types of methylation calls from all sweetpotato samples.

S.N.	Methylation content (%)	Average	Minimum	Maximum
1.	% CpG	45.55	36.75	52.65
2.	%CHG	31.91	27.18	36.02
3.	%CHH	22.54	11.34	36.06
4.	%Hypermethylated	85.41	80.24	89.29
5.	%Hypomethylated	0.76	0.39	1.48
6.	%Intermediate methylated	13.82	10.22	18.56
7.	%Intergenic	84.06	83.17	84.7
8.	%Exonic	6.35	5.82	6.91
9.	%Intronic	9.59	9.26	9.98

### 3.2 Genotypic methylation differences across culture conditions and stress treatments

Genotype specific patterns of DNA methylation were evident in tissue culture and drought stress conditions. The global methylation profiles of three sweetpotato varieties showed distinct genotypic differences under drought conditions. Analysis of drought-induced methylation changes at the gene-level revealed that ‘Murasaki-29’ displayed the largest number of differentially methylated genes (DMGs) in exonic positions per 100-point methylation difference relative to control. Fewer DMGs were detected in ‘Bellevue’ and ‘Bonita’, respectively (**Supplementary Table 5**). Principal component analysis (PCA) of the top 500 most variable exons revealed clear genotype-specific clustering across all three varieties under drought. Tissue culture methylation variability in ‘Bonita’ and ‘Bellevue’ half-sibs showed some overlap as response to subculture stages and ‘Murasaski-29’ half-sibs separated distinctly as a cluster in tissue culture conditions (**Supplementary Figures 3**, **4**). The drought stress treatments showed similar trends; hierarchical clustering of common hypermethylated genes revealed contrasting patterns. ‘Bellevue’ and ‘Bonita’ clustered together, indicating similar epigenetic responses to water deficit. In contrast, clustering patterns for the half-sibs in tissue culture varied (**Supplementary Figures 5**, **6**). ‘Bellevue’ and ‘Murasaki-29’ half-sibs showed a markedly higher number of methylated genes at the final meristem initiated nodal stage. ‘Bonita’ half-sibs exhibited inconsistent performance across all conventional nodal culture stages, whereas ‘Murasaki-29’ and ‘Bellevue’ half-sibs displayed near-uniform patterns throughout the same stages. The number of methylated genes for mother plants was comparable between ‘Bonita’ and ‘Murasaki-29’ half-sibs, while ‘Bellevue’ half-sibs exhibited a numerically higher gene number (**Supplementary Table 6**). ‘Bellevue’ variety showed the highest number of unique methylated genes across all drought stress conditions (control, drought and intermediate drought) as compared to ‘Bonita’ and ‘Murasaki-29’ (**Supplementary Table 7**).

### 3.3 Assessment of tissue culture (Meristem initiated nodal culture and conventional nodal culture) induced methylation

**Figure 1:**
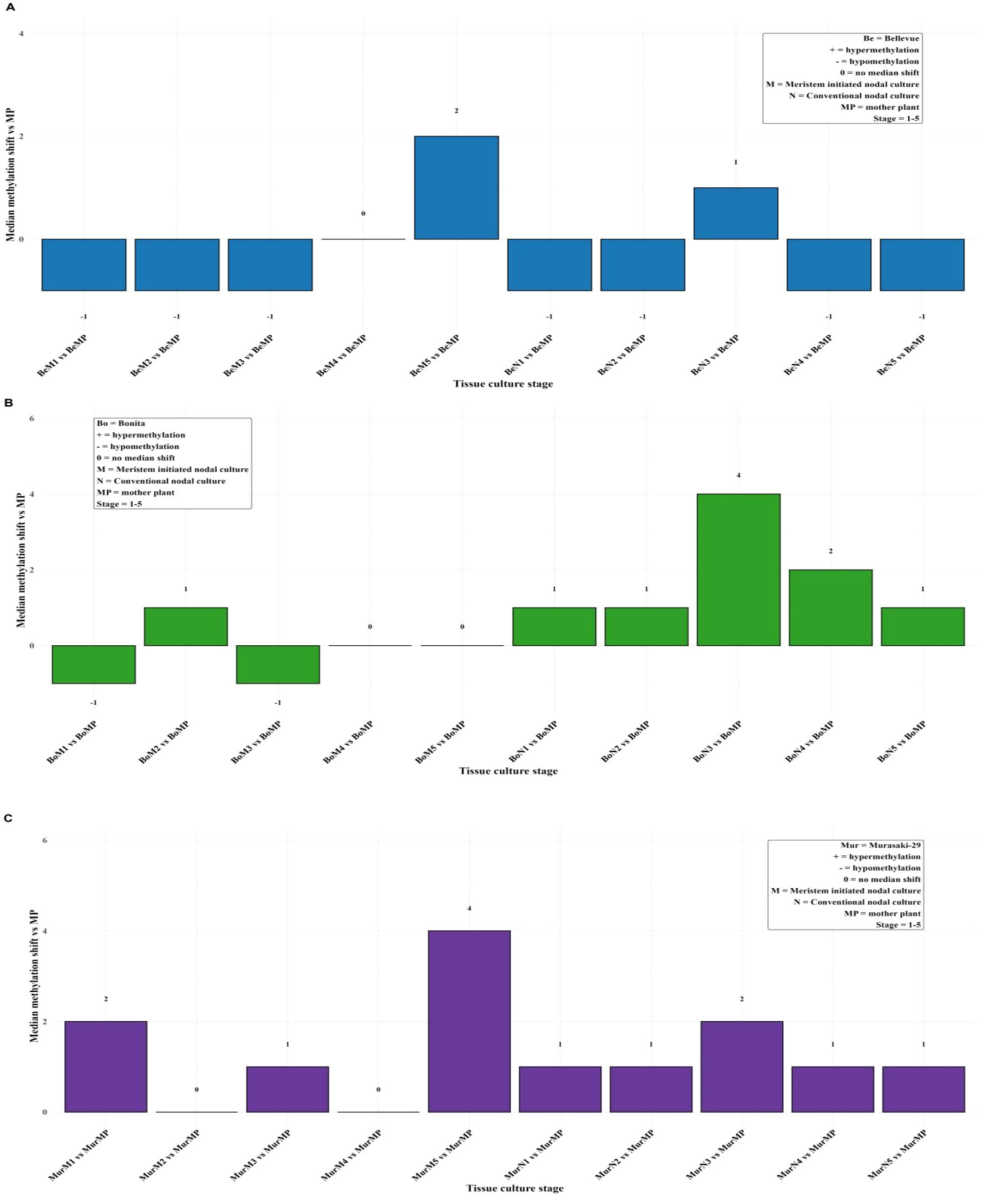
Median methylation shifts across different tissue culture stages in three sweetpotato varieties **(A)** Bellevue, **(B)** Bonita and **(C)** Murasaki-29 relative to the mother plant (MP). Bars represent the median difference in methylation per stage (M = Meristem initiated nodal culture; N = Conventional nodal culture; stages 1–5). Numbers above or below bars indicate the magnitude of the median shift. Statistical significance was assessed using pairwise Wilcoxon signed-rank tests comparing each stage to the mother plant (*p* < 0.05). Positive values (+) indicate hypermethylation, negative values (−) indicate hypomethylation, and 0 indicates no median shift. The top-right legend summarizes the coding for culture type, stage, and methylation change.

Gene-level methylation was quantified across meristematic (M) and nodal (N) stages in ‘Murasaki-29’, ‘Bonita’, and ‘Bellevue’ half-sibs using the mother plant (MP) as the reference. Genes with methylation in at least one condition were retained, and paired Wilcoxon signed-rank tests with FDR correction were performed for each stage relative to MP. Across all half-sibs, tissue culture induced subtle genome-wide methylation changes. Early meristematic stages exhibited hypomethylation, followed by methylation recovery in later nodal stages (**Figure 1**, **Supplementary Table 8**). Variability across loci showed similar pattern too (**Supplementary Figure 7**). A one-way ANOVA followed by *post-hoc* Fisher’s least significant difference (LSD) test revealed significant effects among methylation context, propagation stages, and the MP. Across all half-sibs and stages, CG methylation levels (position counts) were consistently higher than CHG and CHH contexts. The M and N cultures exhibited divergent methylation trajectories: in ‘Bellevue’, half-sibs meristem cultures showed a progressive increase from M1 (30.45%) to M5 (36.7%), while N cultures peaked prematurely at N3 (33.14%). In ‘Bonita’ half-sibs, meristem cultures exhibited a progressive increase in growth from M1 (29.35%) to M4 (33.07%), whereas conventional nodal cultures showed same pattern as ‘Bellevue’ at N3 (37.67%) stage. Conversely, ‘Murasaki-29’ half-sibs displayed high variation across all contexts as compared to half-sibs from other two varieties (**Supplementary Table 9**). Across half-sibs from both varieties, mother plants maintained intermediate methylation levels that differed significantly from both early (M1/N1) and terminal (M5/N5) subculture stages (**Supplementary Figure 8**, **Supplementary Table 10**). Descriptive statistics further support these findings; levels of methylated genes (specifically on exons) were high at the first subculture and remained stable through the fourth stage (4-week interval). However, they increased sharply by the fifth and final stage (6-week interval) in both the ‘Murasaki-29’ and ‘Bellevue’ half-sibs progenies (**Supplementary Table 9**). In contrast, ‘Bonita’ maintained a lower and more uniform methylation pattern across subcultures. Based on percentage change analyses, Meristem initiated nodal cultures exhibited minimal methylation variation up to four weeks, indicating relative stability, whereas conventional nodal cultures displayed greater fluctuation throughout all stages. When the mother plant (MP) was used as the baseline, tissue culture stages showed a moderate overall variability in methylated gene number for all half-sibs (SD = 5,827.92). During the first four nodal subculture cycles (4-week period), Meristem initiated plants in tissue culture exhibited lower variability relative to MP (SD = 4,448.55) than conventional nodal cultures (SD = 5810.45). However, when subjected to extra stress, Meristem initiated tissue culture plants showed elevated methylation divergence at final subculture (6 weeks) in comparison to conventional nodal culture plants (**Supplementary Table 11**).

**Figure 2:**
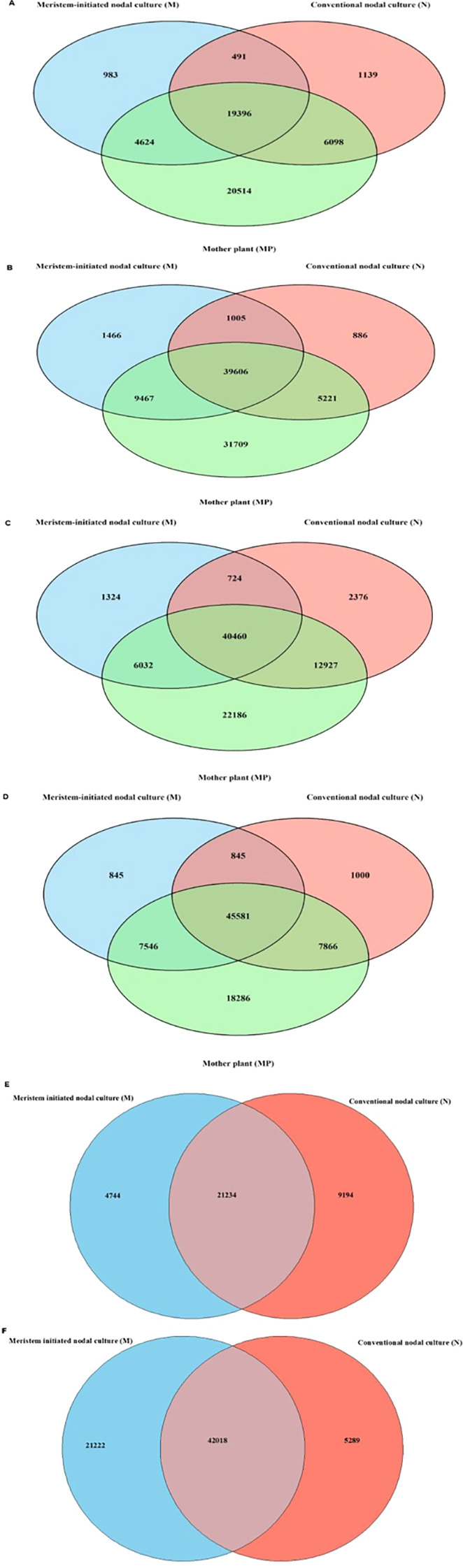
Venn diagram showing the overlap of differentially methylated genes across tissue culture stages in half-sibs of three sweetpotato varieties. **(A)** Combined analysis across all varieties showing overlap between Meristem-initiated nodal culture, conventional nodal culture, and mother plant. Half-sib specific comparisons for **(B)** Bellevue, **(C)** Bonita and **(D)** Murasaki-29. Venn diagram comparing differentially methylated genes between Meristem initiated nodal culture and Conventional nodal culture at **(E)** 4-week and **(F)** 6-week subculture cycle across all varieties.

A total of 19,396 methylated genes was found common among M, N and MP across all half-sibs. Meristem initiated nodal culture showed lowest number of unique methylated genes (983) followed by conventional nodal culture (1,139) and mother plant (20,514) for all half-sibs. Additionally, the number of genes shared across all half-sibs (excluding mother plant) was low in both tissue culture conditions (M and N) in 4 subculture stage (4 weeks) as compared to the final subculture stage (6^th^ week) (**Figure 2**, **Supplementary Table 7**). Gene annotations of DMGs provided information on few high confidence candidates with direct association to tissue culture stress included: *Ibat.Brg_v3.08BG006820.1* encoding a leucine-rich repeat receptor-like protein kinase (LRR-RLK), functioning in somatic embryogenesis competence and cell fate reprogramming; *Ibat.Brg_v3.15DG07340.1* encoding a wall-associated kinase (WAK), mediating cell wall integrity sensing under oxidative stress; and *Ibat.Brg_v3.8CG002980.1* encoding cellulose synthase-like D3 (CSLD3), involved in cell wall biosynthesis during regeneration. Moderately associated genes included *Ibat.Brg_v3.04DG019380.1* (pectin methyl esterase inhibitor), regulating cell wall remodeling during differentiation; *IbatBrg_v3.11FG010280.1* (fatty acid hydroxylase), mediating membrane adaptation; and *Ibat.Brg_v3.06Fg023860.1* (acetyl-CoA carboxylase), controlling carbon allocation. Supporting genes with general stress functions included *Ibat.Brg_v3.02AG010500.1* (protein kinase) for signal transduction and *Ibat.Brg_v3.03BG011760.1* (WD40 repeat protein) for regulatory scaffolding.

### 3.4 Methylation landscape under drought stress

**Figure 3:**
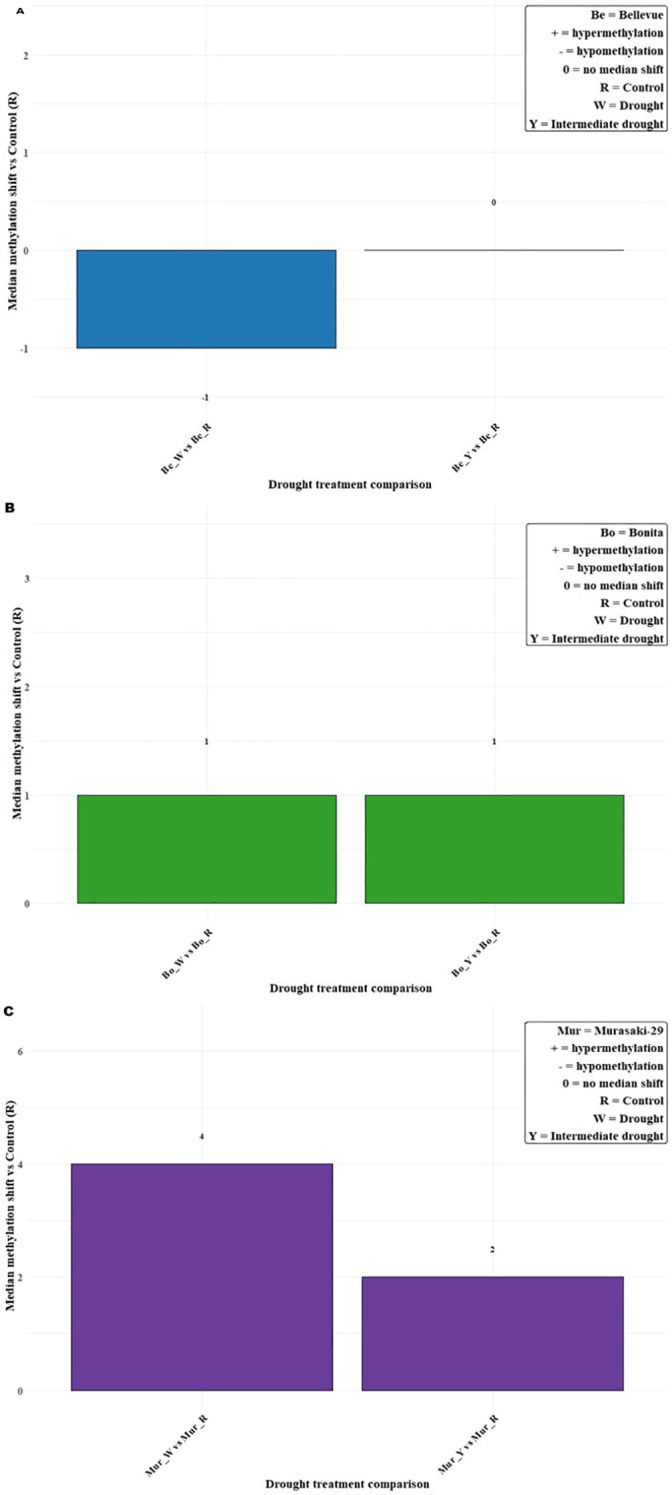
Median methylation shifts under drought treatments in three sweetpotato varieties **(A)** Bellevue, **(B)** Bonita and **(C)** Murasaki-29 relative to the control (R). Bars represent the median difference in methylation under drought treatments (drought; intermediate drought) relative to control (R). Numbers above or below bars indicate the magnitude of the median shift. Statistical significance was assessed using pairwise Wilcoxon signed-rank tests comparing each stage to the control (*p* < 0.05). Positive values (+) denote hypermethylation, negative values (−) denote hypomethylation, and 0 indicates no median shift. The top-right legend shows the coding for treatments and methylation changes.

Gene-level methylation was measured under drought and intermediate drought conditions, using control (non-drought stressed) plants as the reference. Median differences were used to quantify the magnitude and direction of genome-wide methylation shifts, retaining only genes methylated in at least one condition (**Figure 3**; **Supplementary Table 8**). In ‘Bellevue’, median value shifted from -1 to 0, indicating relative methylation stability. ‘Bonita’ exhibited hypermethylation (+1) under both intermediate drought and drought conditions. In contrast, ‘Murasaki-29’ showed the strongest response, with an increase in hypermethyaltion during intermediate drought (Median_diff = +2) and pronounced hypermethylation under drought conditions (Median_diff = +4). This pattern was consistent in variability across loci too (**Supplementary Figure 9**). Moreover, descriptive analysis of the number of methylated genes (present only on exons) across drought treatments revealed distinct patterns observed under control, drought, and intermediate drought conditions in all sweetpotato varieties. In the ‘Murasaki-29’ line, methylated genes was observed in 74,765 regions under control conditions, 81,791 under intermediate drought and 88,596 under drought, representing a ~19% increase in drought relative to control; in ‘Bonita’, the number of methylated genes was 81,478 under control, 85,212 under intermediate drought, and 85,923 under drought, reflecting a moderate increase of ~ 6%; and in contrast, the ‘Bellevue’ lines showed 91,679 methylated genes were found under the control condition as compared to 86,528 under intermediate drought, and 85,199 under drought, which resulted in a ~7% decrease in methylated genes in exonic regions under drought stress (**Supplementary Table 9**). Additionally, chromosomal hypermethylation (methylated position counts on exonic regions) levels using a one-way ANOVA revealed highly significant effects of treatment group (stress conditions x methylation context) on DNA methylation in all varieties. Post Anova, Fisher’s least significant difference (LSD) test (ɑ = 0.05) showed statistical groupings which were used for visualization, while bar diagrams were displayed separately for each variety (**Supplementary Figure 10**, **Supplementary Table 10**). CG methylation exhibited the greatest variation across treatments, with both ‘Murasaki-29’ and ‘Bellevue’ showing significant differences between control and drought conditions based on LSD groupings. Notably, ‘Murasaki-29’ also showed a significant shift in CHG methylation under drought and control, whereas ‘Bellevue’ did not display such variation in this context. In contrast, ‘Bonita’ remained largely stable for methylation levels across treatments, with significant differences observed only under intermediate drought versus control conditions in the CG context. No significant treatment effects were detected in the CHH context across varieties.

**Figure 4:**
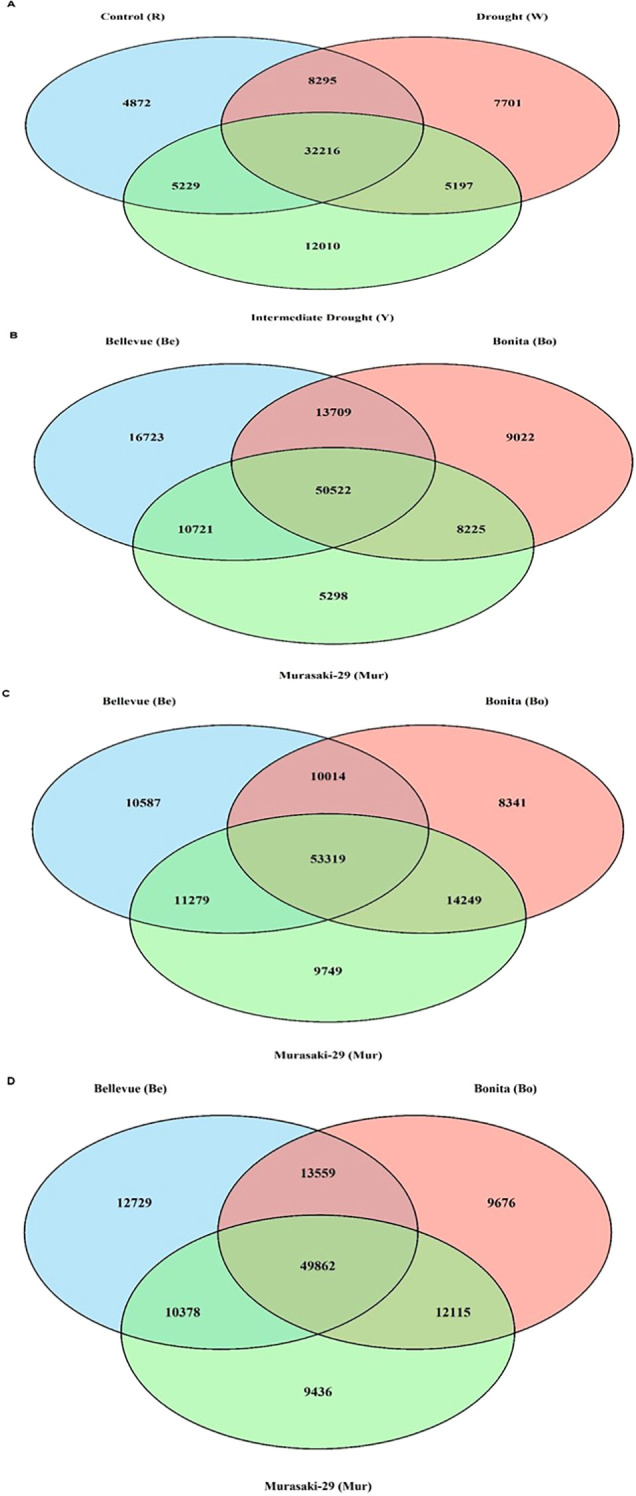
Venn diagrams showing the overlap of differentially methylated genes across drought stress conditions in sweetpotato. **(A)** Combined analysis across all three varieties (Bellevue, Bonita, and Murasaki-29) showing overlap between Control, Drought, and Intermediate drought conditions. Comparison of differentially methylated genes across all three varieties under **(B)** Control, **(C)** Drought, and **(D)** Intermediate drought conditions.

Building on the chromosome-level analysis, we further explored descriptive statistics on common methylated genes across three sweetpotato varieties. A total of 32,126 methylated genes were found common between control, intermediate drought, and drought conditions. The control groups consisted of 4,872, intermediate drought consisted of 12,010 and drought 7,701 uniquely methylated genes, across all varieties. Similarly, evaluation of common methylated genes individually for ‘Bellevue’, ‘Bonita’ and ‘Murasaki-29’ variety; drought condition had 53,319 common genes, intermediate drought consist of 49,862 genes, and the control group consist of 50,522 genes (**Figure 4**; **Supplementary Table 7**). Across all varieties, methylated gene numbers under drought conditions showed a mean standard deviation (SD) of 7,644.55 relative to the control (**Supplementary Table 11**). Gene annotations of DMGs provided information on few high confidence candidates with direct association to drought stress included: *Ibat.Brg_v3.08CG003070.1* encoding Early Responsive to Dehydration 4 (ERD4), involved in immediate dehydration perception and signaling; *Ibat.Brg_v3.13DG014910.1* encoding a Cation/H^+^ exchanger (CAX/NHX family), functioning in osmotic adjustment and ion homeostasis; *Ibat.Brg_v3.08EG008840.1* encoding a Fasciclin-like arabinogalactan protein (FLA), mediating cell wall stability under desiccation stress; and *Ibat.Brg_v3.13AG23150.1* encoding a phosphate transporter 1;5 (PHT1;5), participating in phosphate starvation response that precedes canonical ABA-mediated drought signaling. Moderately associated genes included *Ibat.Brg_v3.13BG018940.1* (protein kinase superfamily), involved in stress signal transduction, and *Ibat.Brg_v3.12AG20830.1* (GNOM-like ARF-GEF), regulating auxin transport and adaptive root architecture modifications.

## 4 Discussion

Two distinct types of stress were considered in this study: *in vitro* stress associated with tissue culture propagation and environmental stress caused by drought treatments (**Supplementary Figure 1**). Unlike drought, which was deliberately imposed as an environmental stress on greenhouse grown plants, *in vitro* stress arises inherently from the artificial conditions of tissue culture. Continuous subculturing and prolonged maintenance in the culture medium can induce physiological and epi(genetic) stress responses even in the absence of external stimuli (Ghosh et al., 2021). Tissue culture has been extensively used for the production and propagation of disease-free planting materials (Sello et al., 2018), especially in vegetatively propagated crops, such as sweetpotato. However, the key challenge is to use these regenerated lines effectively while preserving their epigenetic stability. Our study demonstrates that epigenetic stability represents a complementary component of overall genome integrity. This study focuses on methylation in exonic regions to ensure their direct, interpretable link to gene expression and subsequently phenotype. In plants, gene body methylation is primarily enriched in exons that signals stable gene expression. For complex polyploids such as sweetpotato, an exon-centric approach improves sequence alignment confidence, reduces intergenic noise, and instability Intergenic regions in sweetpotato are comparatively less functionally characterized, particularly given its polyploid and transposable element rich genome, which makes interpretation of methylation changes in these regions more challenging. This is the first genome-wide bisulfite-based methylation profiling in sweetpotato, a high-ploidy crop to map DNA methylation patterns under tissue culture, assess genotypic differences, and an environmental stressor (drought).

The comparative assessment of DNA methylation dynamics under tissue culture and drought stress revealed distinct modes of epigenetic regulation in sweetpotato. Comparing tissue culture and drought-induced methylation changes revealed a clear difference in epigenetic stability. Tissue culture produced consistent, subtle genome-wide changes (Median_diff ±1-2) across all varieties and culture stages, suggesting that the epigenome is largely maintained (**Figure 1**; **Supplementary Figure 7**; **Supplementary Table 8**). The meristematic tip culture, produces disease-free plantlets by stimulating rapid axillary bud growth, which bypasses pathogen-laden vascular systems and avoids the genetic instability of disorganized growth phases (Wondimu et al., 2012). Other tissue culture studies showed locus-specific methylation linked to somaclonal variation in maize regenerants (Stelpflug et al., 2014) and grapes (Schellenbaum et al., 2008). In contrast, drought stress triggered larger and more variable shift, particularly in Murasaki-29 (± 4 under drought conditions), highlighting variety-specific epigenetic response indicative of active epigenetic reprogramming in response to environmental pressure (**Figure 3**; **Supplementary Figure 9**; **Supplementary Table 8**). These differentially methylated exonic responses suggest that cytosine methylation in sweetpotato is dynamically regulated under drought, functioning as a component of adaptive stress signaling and transcriptional modulation (Sun et al., 2022). Similar patterns of methylation under drought stress have been reported in cotton (Lu et al., 2017) and mulberry (Li et al., 2020). There was a higher number of commonly methylated genes across control, drought, and intermediate drought treatments, together with larger median genome-wide shifts, indicating coordinated and robust epigenetic reprogramming associated with environmental stress (**Figures 2**, **4**; **Supplementary Table 7**). Statistical analysis of chromosome-wise hypermethylated positions on exonic region showed intensity-dependent shifts in methylation level from control through intermediate drought and drought with high group effects and stepwise LSD separation particularly in ‘Murasaki-29’ indicating strong and active epigenetic reprogramming in response to water deficit. However, across meristem (M1-M5) and nodal (N1-N5) subculture stages, methylation levels showed minor fluctuation around mother plant (MP) baseline, with significantly smaller sizes (**Supplementary Figures 8**, **10**; **Supplementary Table 10**). The absence of cumulative drift across subcultures demonstrates strong epigenetic homeostasis and preserves overall stability under *in vitro* conditions (Ghosh et al., 2021; Sun et al., 2022).

A notable observation from this study was the temporal pattern of methylation changes across propagation stages. Meristem culture avoids the dedifferentiation of cells by using pre-existing, organized stem cells, which maintain high levels of CHG and CHH methylation to protect genome integrity and keep “parasitic” sequences silenced. These stem cells undergo reduced cell division rates compared to rapidly dividing callus cells, thereby minimizing the rounds of replication and reducing the risk of spontaneous mutations and replication-related epigenetic drift. Furthermore, they consist of specialized small RNA pathways that specifically target and silence transposable elements (TEs), preventing parasitic genetic elements from disrupting the epigenomic landscape (Nguyen and Gutzat, 2022). The methylation level along with number of methylated genes increased markedly during the first week compared with the mother plant in our study, known as tissue culture induced variation (TCIV), likely reflecting an initial phase of epigenetic reprogramming as the explants adjusted to *in vitro* culture conditions (**Supplementary Tables 6**, **9**) (Bednarek and Orłowska, 2020; Ghosh et al., 2021). This is further supported by the gene annotation which identified DMGs spanning somatic embryogenesiscompetence (LRR-RLK), cell wall integrity sensing (WAK), structural biosynthesis (CSLD3), wall remodeling (pectin methyl esterase inhibitor), and membrane adaptation (fatty acid hydroxylase) reveals that epigenetic regulation orchestrates a coordinated response to oxidative stress inherent in tissue culture conditions potentially enabling cells to balance between structural rigidity for stress tolerance and flexibility required for cell programming. Following this early response, methylation levels remained relatively stable through the fourth subculture in meristem initiated nodal culture as compare to conventional nodal culture, suggesting that once cellular homeostasis was established, the epigenetic landscape became stabilized to support sustained growth. However, a sharp increase in methylation level and DMGs by the 5th stage (4^th^ week vs. 6^th^ week subculturing) was observed in some varieties, coinciding with prolonged culture duration and the potential accumulation of *in vitro*–induced stress, such as oxidative stress, nutrient depletion, or hormonal imbalance (**Supplementary Figures 8**, **10**; **Supplementary Table 9**). This late stage increase in methylation may represent an adaptive response aimed at maintaining genome integrity under extended tissue culture stress. Propagations stages for conventional nodal culture were rather inconsistent and fluctuated in first four stages as compared to meristem initiated nodal culture. Nevertheless, compared to the mother plant, subcultured lines (in both meristem initiated and conventional nodal culture) can acquire subtle epigenetic changes and less chance of transferring epigenetic changes to subsequent generation (Ghosh et al., 2021). Therefore, sourcing explants from mother plants under optimal greenhouse conditions and maintaining consistent subculturing practices are critical for minimizing epigenetic variability. In contrast, under drought conditions, the methylation landscape exhibited a different trajectory. Despite full rehydration prior to sampling, the methylation profiles of drought-treated plants did not revert to those of control group in any variety (**Supplementary Tables 6**, **S9**). This incomplete recovery suggests that drought-induced methylation events may persist even after the stress is alleviated, implying the presence of stable epigenetic imprinting (Sun et al., 2022). The continuous six-month tissue culture process facilitated the progressive establishment of mitotically stable methylation events (Ghosh et al., 2021; Nguyen and Gutzat, 2022). Conversely, drought, primarily elicited non-cumulative methylation changes and epigenetic reprogramming underscoring a key divergence in the stability of induced epigenetic responses (**Supplementary Figures 8**, **10**: **Supplementary Table 10**). This pattern aligns with findings that abiotic/environment stresses, particularly drought, induce widespread DNA methylation changes to regulate stress-responsive genes (Sun et al., 2022). One of the reasons for this phenomenon could be the highly diverse proteins involved in regulation of these epigenetic variations and mechanism called priming where plants adapt to prepare for future stress (Kambona et al., 2023; Lämke and Bäurle, 2017; Sun et al., 2022). Such persistent methylation changes have been reported in crops such as wheat where prior stress exposure enhances tolerance against future stress (Kambona et al., 2023). Moreover, gene annotations help identify DMGs spanning direct drought response (ERD4), osmotic regulation (CAX/NHX), structural integrity (FLA), early nutrient sensing (PHT1;5), stress signaling (protein kinase), and hormone-mediated growth plasticity (GNOM-like) suggests that epigenetic regulation coordinates a multi-layered adaptive response in sweetpotato potentially enabling preemptive stress adaptation before severe water deficit occurs. Hence, these findings highlight that while tissue culture may trigger transient epigenetic adjustments during early adaptation, it largely preserves epigenetic stability and methylation stability, unlike drought stress, which induces both acute and permanent epigenetic reprogramming/modifications associated with stress memory rather than recovery (Sun et al., 2022). This interpretation was further supported by our study with the detection of greater methylation variability under drought stress as compared to tissue culture (**Supplementary Table 11**). This stark difference reinforces that tissue culture maintains epigenetic stability, while drought stress may involve transcription factors (TFs) inferring enzymatic activities (such as methyltransferase and demethylase) and induces coordinated methylation remodeling across numerous stress-related genes, potentially linked to regulatory pathways governing water deficit responses (Kambona et al., 2023; Lämke and Bäurle, 2017). Collectively, these findings highlight a clear epigenetic dichotomy between tissue culture and drought environments.

Tissue culture provides a controlled, low-stress system that sustains genomic and methylation stability, making it an optimal system for the long-term preservation and propagation of clonally derived genotypes (Nguyen and Gutzat, 2022; Villordon and LaBonte, 1996). Environmental stress such as drought, on the other hand, destabilizes methylation patterns, activating extensive epigenetic remodeling as part of the plant’s adaptive mechanism (Sun et al., 2022). While such plasticity may be beneficial for stress tolerance and resilience, it introduces variability that may affect gene expression consistency and heritable epigenetic states. Therefore, from a methylation maintenance perspective, *in vitro* propagation provides a highly stable epigenetic environment, maintaining methylation homeostasis and minimizing stochastic reprogramming (**Figures 1**, **3**; **Supplementary Table 8**). On the other hand, *in vivo* stressor events like drought, are common occurrences in a greenhouse environment. Hence, tissue culture can be considered as a reliable method for clonal propagation and germplasm conservation, preserving the original identity of elite parental lines across multiple subcultures (Ghosh et al., 2021). A previous research by Akomeah et al. (2019) demonstrated that micropropagation in sweetpotato induces significant epigenetic alterations with reductions in key nutritional traits and accounting for up to 29% of trait variation. However, their study utilized the selective methylation sensitive amplification polymorphism, which may not have captured the methylation islands at a whole genome scale. Our study employed whole-genome bisulfite sequencing to provide a higher-resolution view of genome-wide DNA methylation dynamics in half-sib progenies and varieties of sweetpotato subjected to both tissue culture (meristem initiated vs. conventional nodal) and drought stress, respectively. Unlike Akomeah et al. (2019) that focused on nutritional consequences, our work identifies specific temporal and genotype-specific windows of epigenetic fluctuation, underscoring the need for fine-tuning propagation timelines to safeguard epigenetic stability. Together, these studies emphasize the importance of integrating epigenetic monitoring into sweetpotato breeding and propagation programs to minimize somaclonal variation and maintain epigenetic integrity.

### 4.1 Epigenetic regulation is genotype-dependent

Epigenetic regulation in sweetpotato was strongly dependent on both drought (environmental stress) and tissue culture conditions, reflecting variety-specific differences in methylation, plasticity and stability. Under drought, ‘Bellevue’ exhibited a higher number of methylated genes (present only on exons) under control conditions than under stress (drought and intermediate drought) (**Supplementary Table 6**), suggesting the activation of DNA demethylases (dMTases), as reported in rice (Wang et al., 2016). In contrast, ‘Bonita’ and ‘Murasaki-29’ displayed higher methylated genes under drought, consistent with increased DNA methyltransferase (C5-MTase) activity and stress-induced hypermethylation (**Figure 3**). Similar differences were observed when genome-wide comparisons between ‘indica’ and ‘japonica’ rice accessions revealed extensive differentially methylated regions associated with subspecies divergence (Yan et al., 2025). ‘Murasaki-29’ responds to drought stress with a greater number of differentially methylated genes (based on exonic positions) compared with ‘ Bellevue ‘ and ‘Bonita’, highlighting potential varietal differences in the regulation of stress-responsive genes and suggesting that ‘Murasaki-29’ may possess higher epigenetic flexibility under water-deficit conditions (**Supplementary Table 5**).

‘Murasaki-29’ half-sibs retained the highest number of common methylated genes across various tissue culture conditions (M, N and MP), followed sequentially by ‘Bonita’ and ‘Bellevue’ half-sibs (**Figure 2**; **Supplementary Table 8**). In summary, these findings highlight that ‘Murasaki-29’ is an epigenetically flexible “fast learner”, capable of extensive reprogramming under drought and *in vitro* stress. In contrast, ‘Bellevue’ maintains a conservative, stable methylome, while ‘Bonita’ exhibits intermediate plasticity. This divergence underscores that the genotypes possess fundamentally different “toolkits” for epigenetic adaptation (Wang et al., 2016; Yan et al., 2025). A similar study in comparative methylome analyses across diverse inbred maize lines demonstrated genotype-specific methylation variation linked to gene expression differences (Xu et al., 2020). Distinct clusters displayed by the PCA and heatmap supported these patterns, in both tissue culture and drought studies suggesting that individual plant genetic background plays an important role in overall epigenetic phenomenon i.e. broad methylation stability, flexibility, and stronger epigenetic plasticity (**Supplementary Figures 3**–**6**) (Ghosh et al., 2021; Sun et al., 2022). It should be noted that sweetpotato varieties differ substantially in their genomic composition, transposable element load, and basal methylation patterns due to their distinct breeding origins and high polyploidy (Akomeah et al., 2019; Yang et al., 2025). As a result, each variety carries a unique epigenetic configuration and responds differently to environmental or *in vitro* stress. Hence, these findings reinforce that epigenetic plasticity is an intrinsic, variety-dependent trait, likely governed by genetic background and enzyme activity balance (for example, C5-MTase vs dMTase). Understanding such genotype-specific methylation dynamics provides a valuable framework for identifying varieties with enhanced adaptability or genomic stability.

## 5 Conclusion

Our study provides a comprehensive profile of differential methylation between sweetpotato varieties and culture conditions. This study is critical to understand how epigenetic responses to drought and tissue culture stress can affect epigenetic stability in clonally propagated sweetpotato varieties, informing better strategies for long-term germplasm preservation. The results suggest tissue culture as a more reliable way of long-term maintenance given environmental variations found in greenhouses or *in vivo* culture. It also highlights dynamic, genotype-specific methylation changes in sweetpotato under drought and tissue culture stress. Propagation methods and explant variety (genotype) significantly impacted epigenetic stability, an important component of genetic fidelity.

These findings support integrating epigenetic monitoring into propagation strategies to safeguard elite germplasm integrity. While the present study discussed a small fraction of methylation patterns focusing only on exonic regions, our future endeavors will include intergenic and intronic methylations that will provide comprehensive view of the methylation dynamics as they relate to the epigenetic stability of sweetpotato. Future work needs to integrate phenotypic assessments under drought and *in vitro* conditions, together with gene expression profiling, to clarify the functional impact of these methylation changes. Integrating epigenetic, transcriptomic, and physiological data will enhance understanding of the biological significance of these epigenetic modifications in maintaining epigenetic stability in sweetpotato.

## Data Availability

The datasets presented in this study can be found in online repositories. The names of the repository/repositories and accession number(s) can be found below: https://zenodo.org/records/18559551, 10.5281/zenodo.18559551.
