# Supplementary material for: Maintaining genetic stability in sweet potato: epigenetic insights into propagation and drought tolerance
Source: Front Plant Sci. 2026 May 5;17:1807723. doi: 10.3389/fpls.2026.1807723 (PMC13184602; doi:10.3389/fpls.2026.1807723)
Supplement: Supplementary file 2 [file Table2.docx]

**Supplementary Table 2** **Greenhouse temperature and relative humidity data for tissue culture and drought stress study.**

| **S.N.** | **Experiment/study** | **Time** | **Temperature (°C)** | **Relative Humidity (%)** |
| --- | --- | --- | --- | --- |
| 1. | Drought study | Day | 33.2 ± 4.0 | 64.2 ± 12.8 |
|  |  | Night | 25.8 ± 1.7 | 87.4 ± 7.2 |
| 2. | Mother plant (Tissue culture study) | Day | 27.9 ± 5.1 | 57.9 ± 42.1 |
|  |  | Night | 23.4 ± 3.8 | 61.4 ± 38.2 |

**Mean±SD**
