# Supplementary material for: Maintaining genetic stability in sweet potato: epigenetic insights into propagation and drought tolerance
Source: Front Plant Sci. 2026 May 5;17:1807723. doi: 10.3389/fpls.2026.1807723 (PMC13184602; doi:10.3389/fpls.2026.1807723)
Supplement: Supplementary file 4 [file Table4.docx]

**Supplementary Table 4 Descriptive statistics of reads before and after filtration.**

| **S.N.** | **Alignment Statistics** | **Raw/Unfiltered** | **Filtered** |
| --- | --- | --- | --- |
| 1. | Sequencing reads | 100 % | 94.95 % (average across all samples) |
| 2. | Cytosines count | 985,099,340 (36.52%) | 117,065,070 (11.88%) |
| 3. | Scaffolds | 6629 | 2672 |
| 4. | Mapping efficiency | 100 % | 33.8 % (average across all samples) |
| 5. | Cytosine methylated in unknown context | 100 % | 18.82 % (average across all samples) |
| 6. | Methylated cytosine on plus strand (+) | 117,065,070 | 58,555,260 (50.02%) |
| 7. | Methylated cytosine on minus strand (-) | 117,065,070 | 58,509,810 (49.98%) |
