# Supplementary material for: Maintaining genetic stability in sweet potato: epigenetic insights into propagation and drought tolerance
Source: Front Plant Sci. 2026 May 5;17:1807723. doi: 10.3389/fpls.2026.1807723 (PMC13184602; doi:10.3389/fpls.2026.1807723)

**Supplementary Figures:**

**Supplementary Figure S1** Schematic representation of tissue culture and drought study across different propagation methods, drought treatments and varieties.

6 week

4 week

4 week

4 week

**Meristem induced nodal culture**

M5

M4

M3

M2

M1

**Motherplant**

**Bellevue Half sibs**

MP

**Conventional nodal culture**

N4

N3

N5

N2

N1

6 week

4 week

4 week

4 week

**Meristem induced nodal culture**

M5

M4

M3

M2

M1

**Motherplant**

**Bonita Half sibs**

MP

**Conventional nodal culture**

N4

N3

N5

N2

N1

6 week

4 week

4 week

4 week

6 week

4 week

4 week

4 week

**Meristem induced nodal culture**

**Motherplant**

M5

M4

M3

M2

M1

**Murasaki-29 Half sibs**

MP

**Conventional nodal culture**

N4

N3

N5

N2

N1

6 week

4 week

4 week

4 week

**Drought Study**

**7 DAYS**

**14 DAYS**

**Bellevue main variety**

**Bonita main variety**

**Murasaki-29 main variety**

**0 DAYS**

CONTROL

DROUGHT

CONTROL

INTERMEDIATE DROUGHT

CONTROL

DROUGHT

INTERMEDIATE DROUGHT

INTERMEDIATERDROUGHT

DROUGHT

**Tissue Culture Study**

**Supplementary Figure S2** Visual representation of tissue culture study for three different half sibs progenies from three main varieties (Bellevue, Bonita and Murasaki-29).


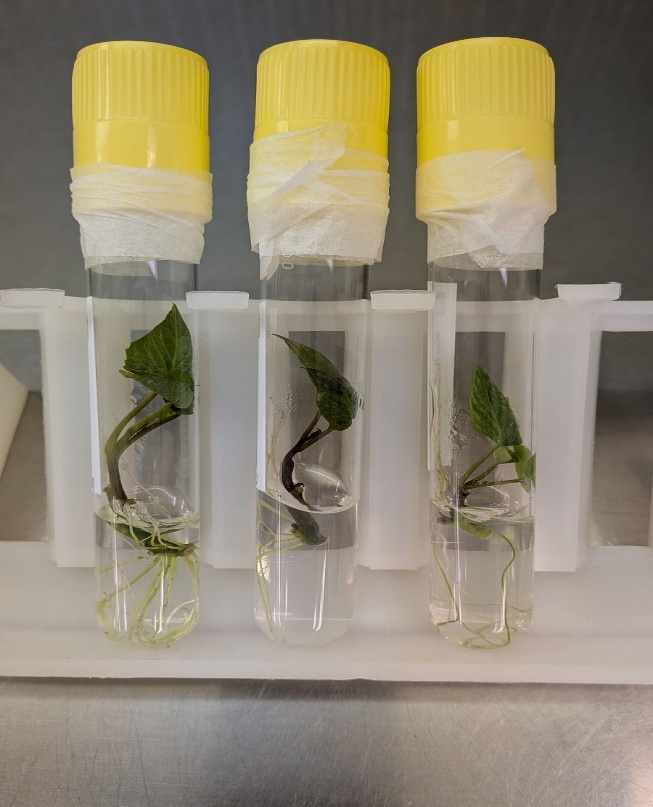

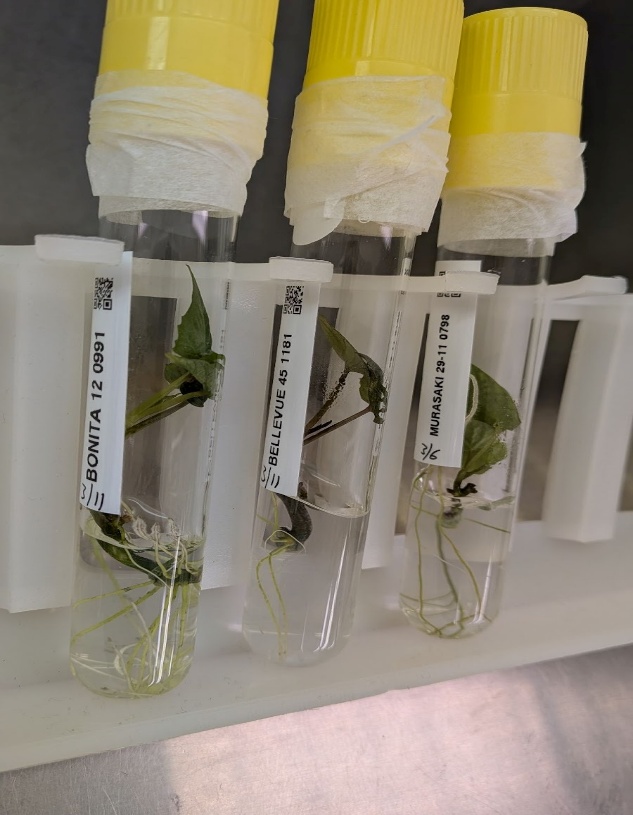


**Supplementary Figure S3** Principal component analysis (PCA) of DNA methylation patterns in sweetpotato tissue culture samples for major DNA methylated exonic regions. The analysis includes three varieties: Bellevue (Be, circles), Bonita (Bo, triangles), and Murasaki-29 (Mur, squares) across three tissue culture stages: M = Meristem initiated nodal culture (blue), N = Conventional nodal culture (green), and MP = Mother Plant (red). PC1 explains 23.91% of the variance and PC2 explains 16.01% of the variance.


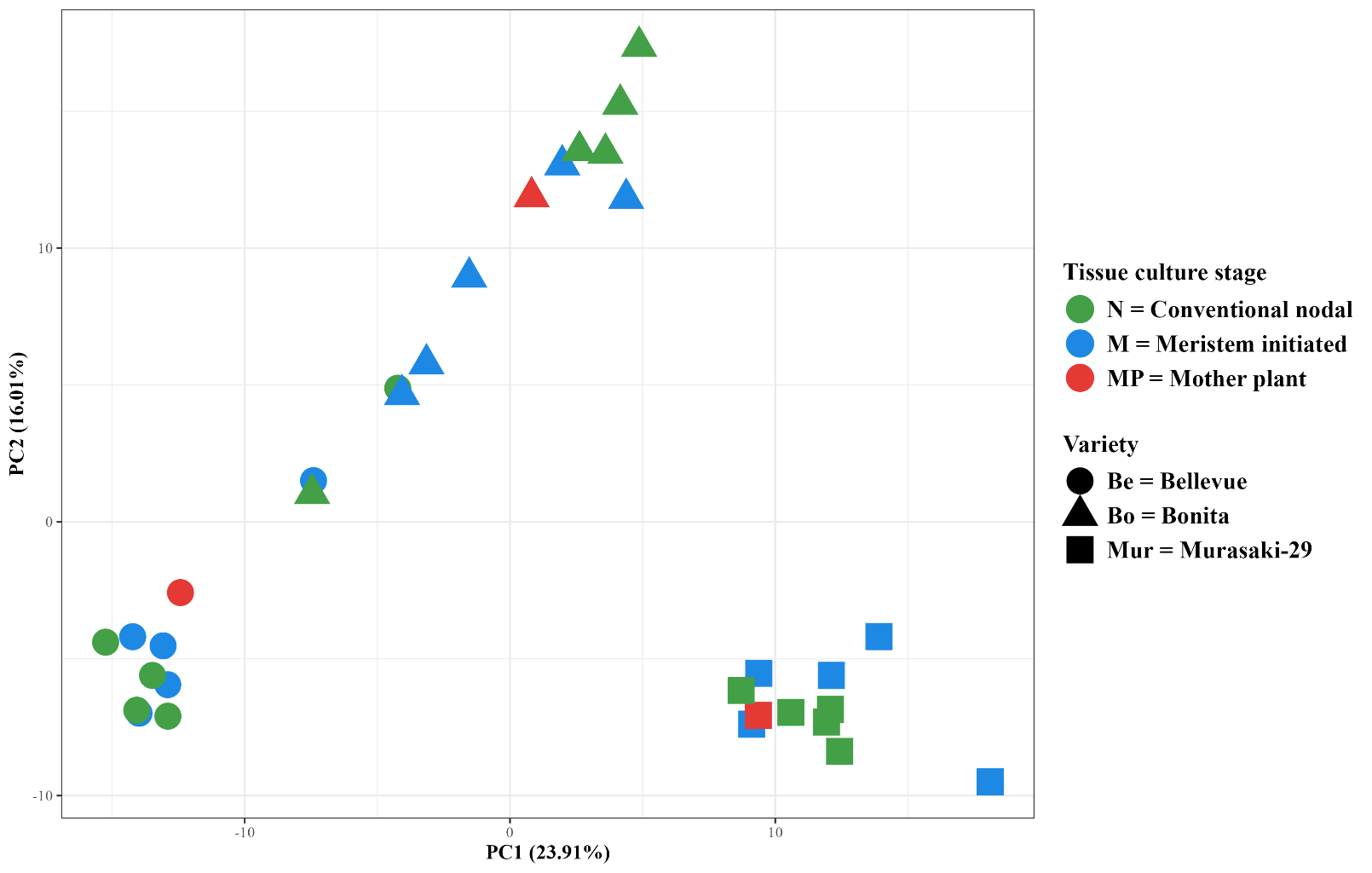


**Supplementary Figure S4** Principal component analysis (PCA) of DNA methylation patterns in sweetpotato varieties under drought stress conditions for major DNA methylated exonic regions. The analysis includes three main varieties: Bellevue (Be, circles), Bonita (Bo, triangles), and Murasaki-29 (Mur, squares) under three water conditions: R = Control (green), W = Drought (red), and Y = Intermediate drought (orange). PC1 explains 28.77% of the variance and PC2 explains 22.27% of the variance.


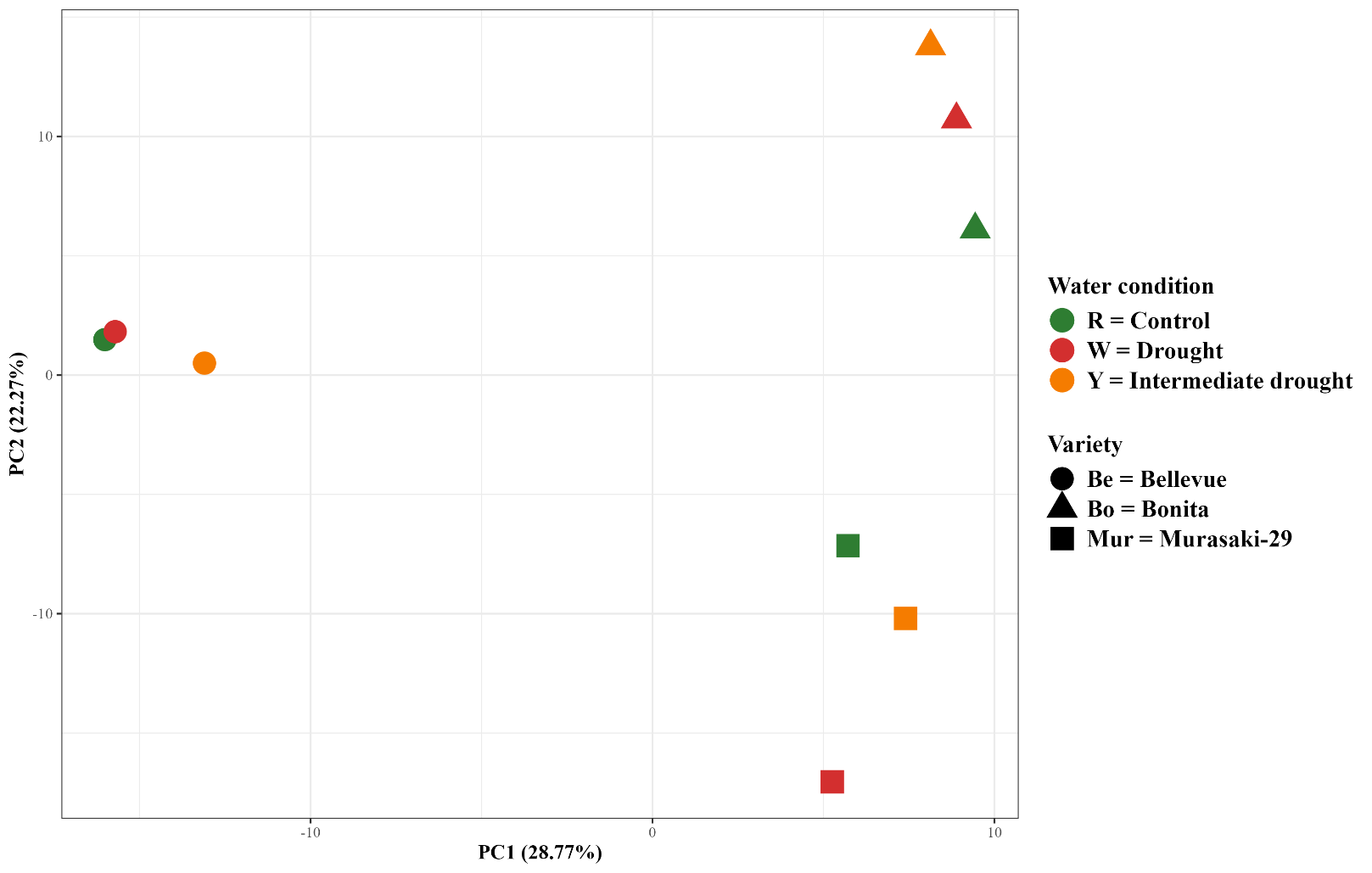


**Supplementary Figure S5** Heatmap showing common patterns of highly DNA methylated regions across tissue culture stages in **(A)** Bellevue, **(B)** Bonita, and **(C)** Murasaki-29 half sibs progenies. Rows represent genes and columns represent samples across developmental stages: M1-M5 = Meristem initiated nodal culture stages 1-5, MP = Mother Plant, and N1-N5 = Conventional nodal stages 1-5. Colors represent row-scaled (Z-score normalized) methylation values. Hierarchical clustering using correlation distance reveals distinct methylation patterns among genes and tissue culture stages.

**A**


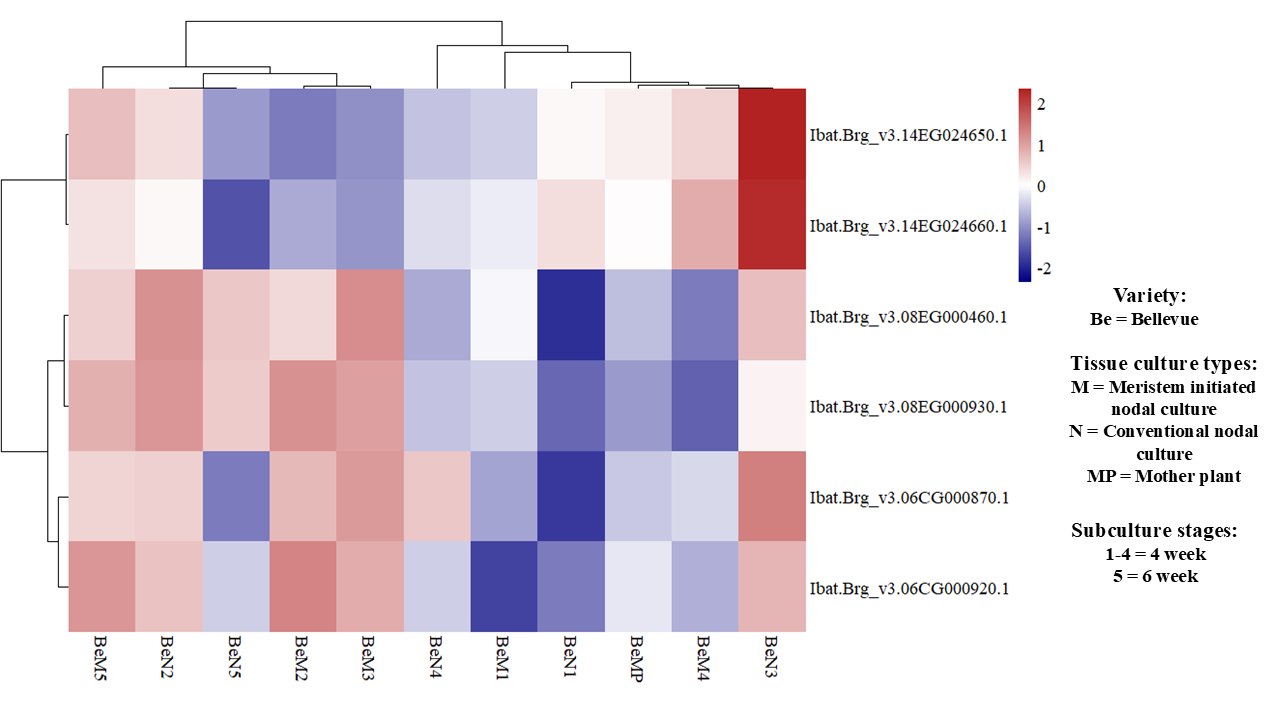


**B**


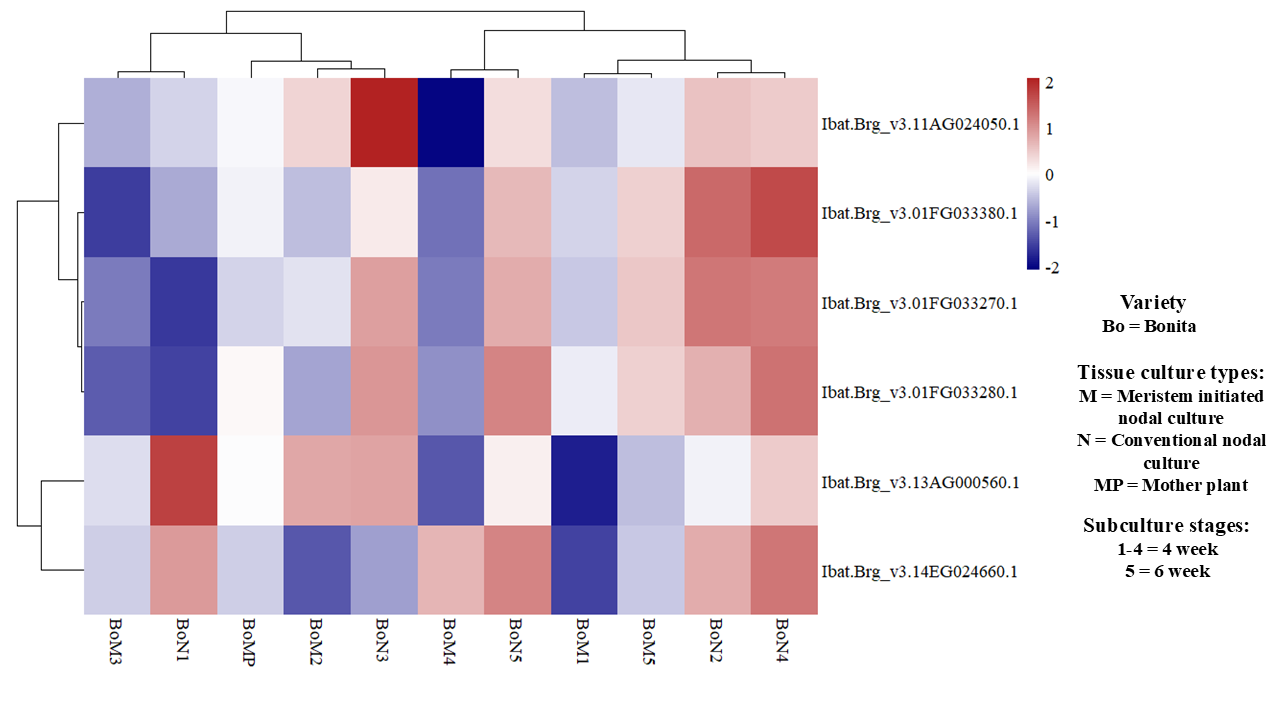


**C**


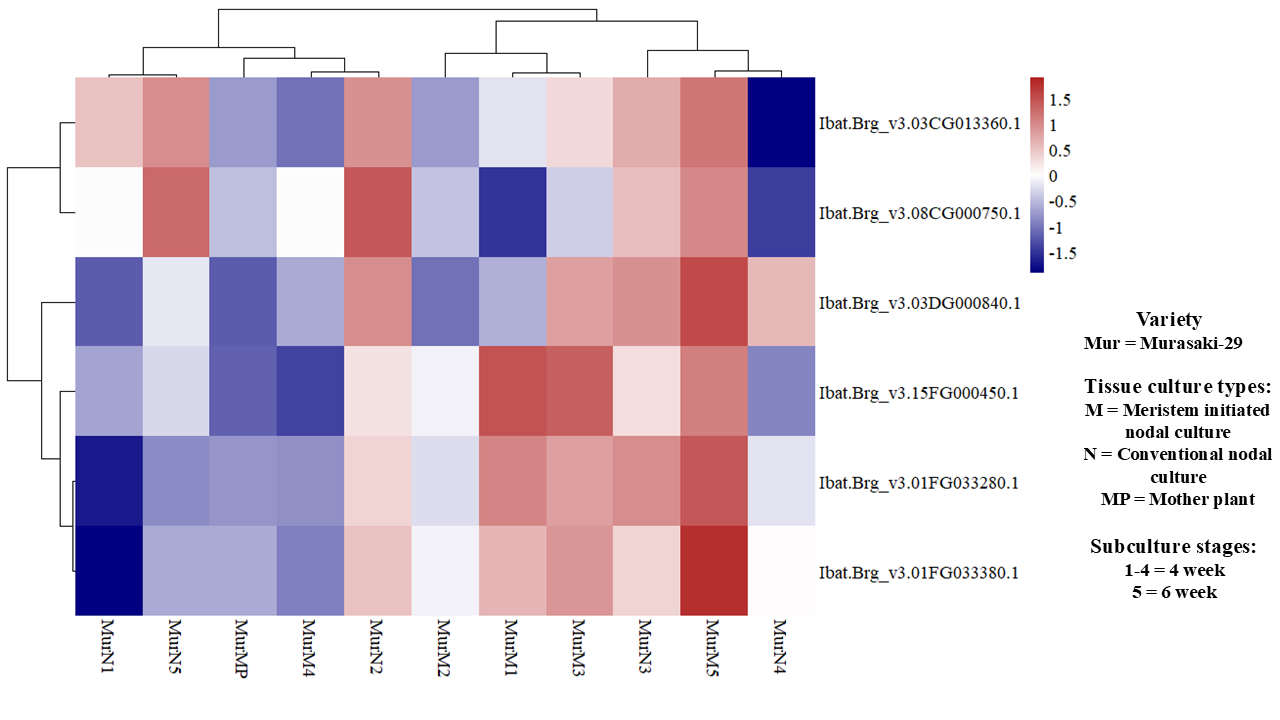


**Supplementary Figure S6** Heatmap showing common patterns of highly DNA methylated regions across drought stress condition in three sweetpotato varieties. Rows represent genes and columns represent samples from three varieties: Bellevue (Be), Bonita (Bo), and Murasaki-29 (Mur) under three drought stress conditions: R = Control, W = Drought, and Y = Intermediate drought. Colors represent row-scaled (Z-score normalized) methylation values. Hierarchical clustering was performed using correlation distance to group genes and drought stages with similar methylation patterns.


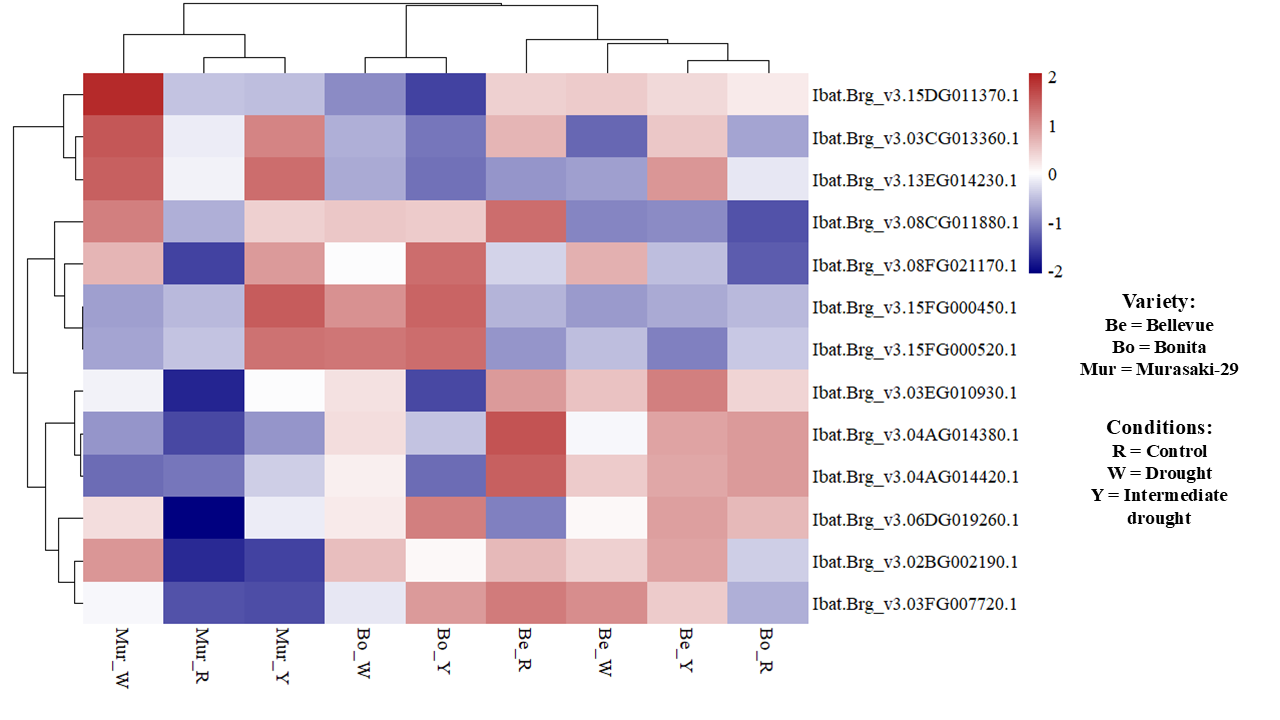


**Supplementary Figure S7** Variability across loci (gene-level) analysis across different tissue culture stages in three sweetpotato half-sibs varieties **(A)** Bellevue (Mother plant and Meristem induced nodal culture stages), **(B)** Bellevue (Conventional nodal culture stages), **(C)** Bonita (Mother plant and Meristem induced nodal culture stages), (**D**) Bonita (Conventional nodal culture stages), (**E**) Murasaki-29 (Mother plant and Meristem induced nodal culture stages) and (**F**) Murasaki-29 (Conventional nodal culture stages). Three different boxplot in different colors each representing different tissue culture are shown. Mean value is given with diamond shape label in center while quartiles are presented outside the boxes. X-axis represent stages of culture while Y-axis represent methyaltion level.

**A**


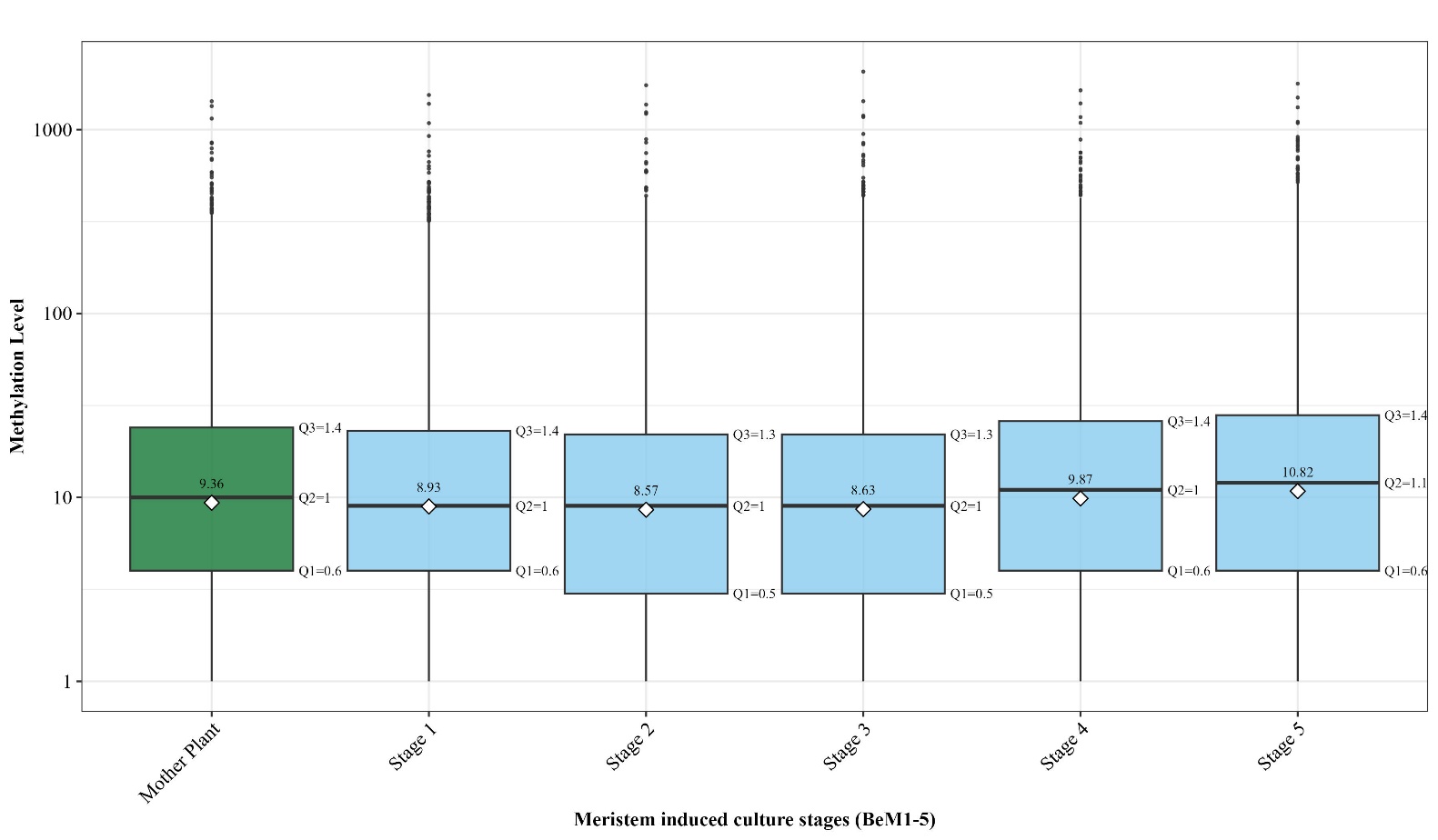


B
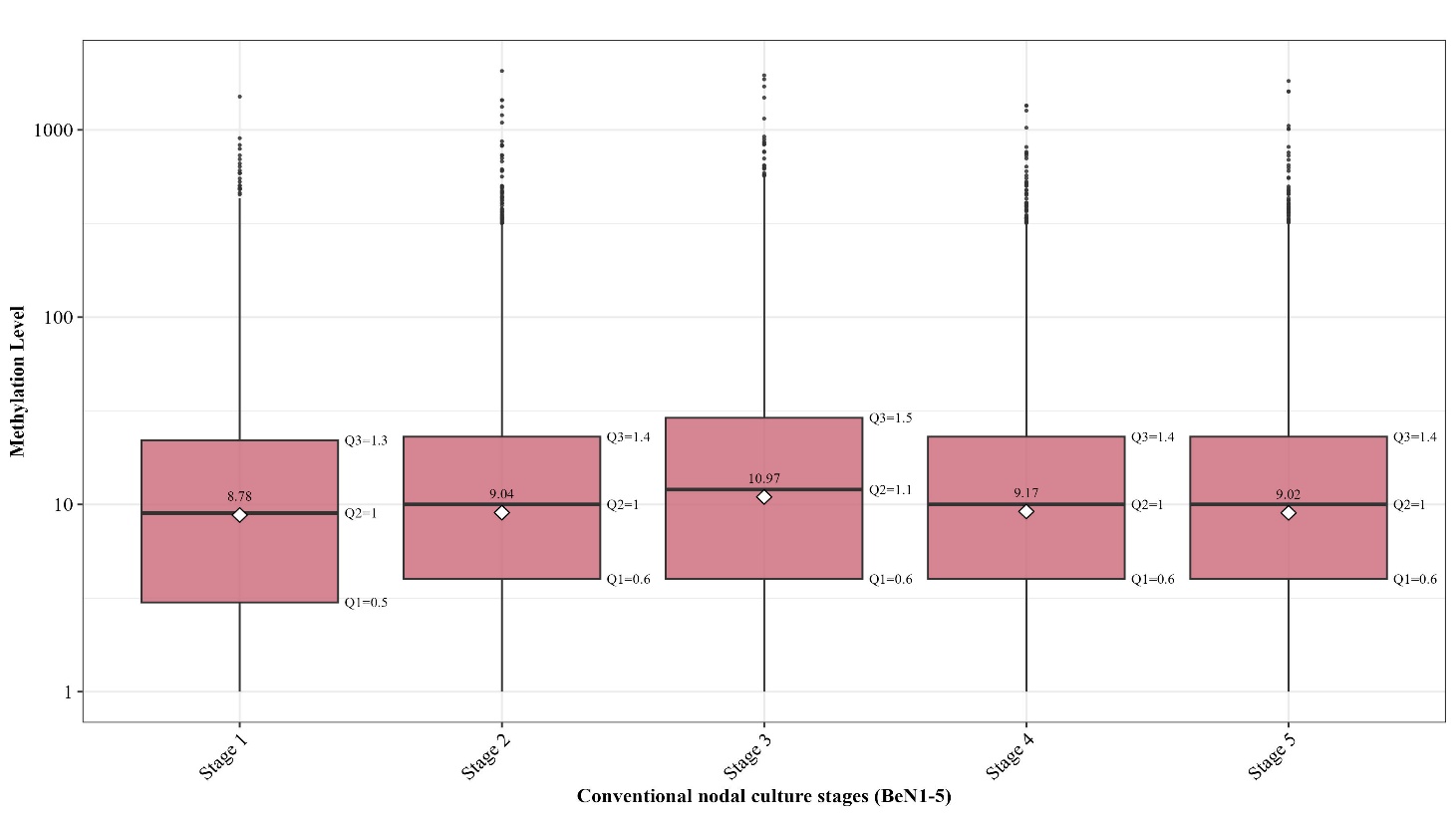


**C**
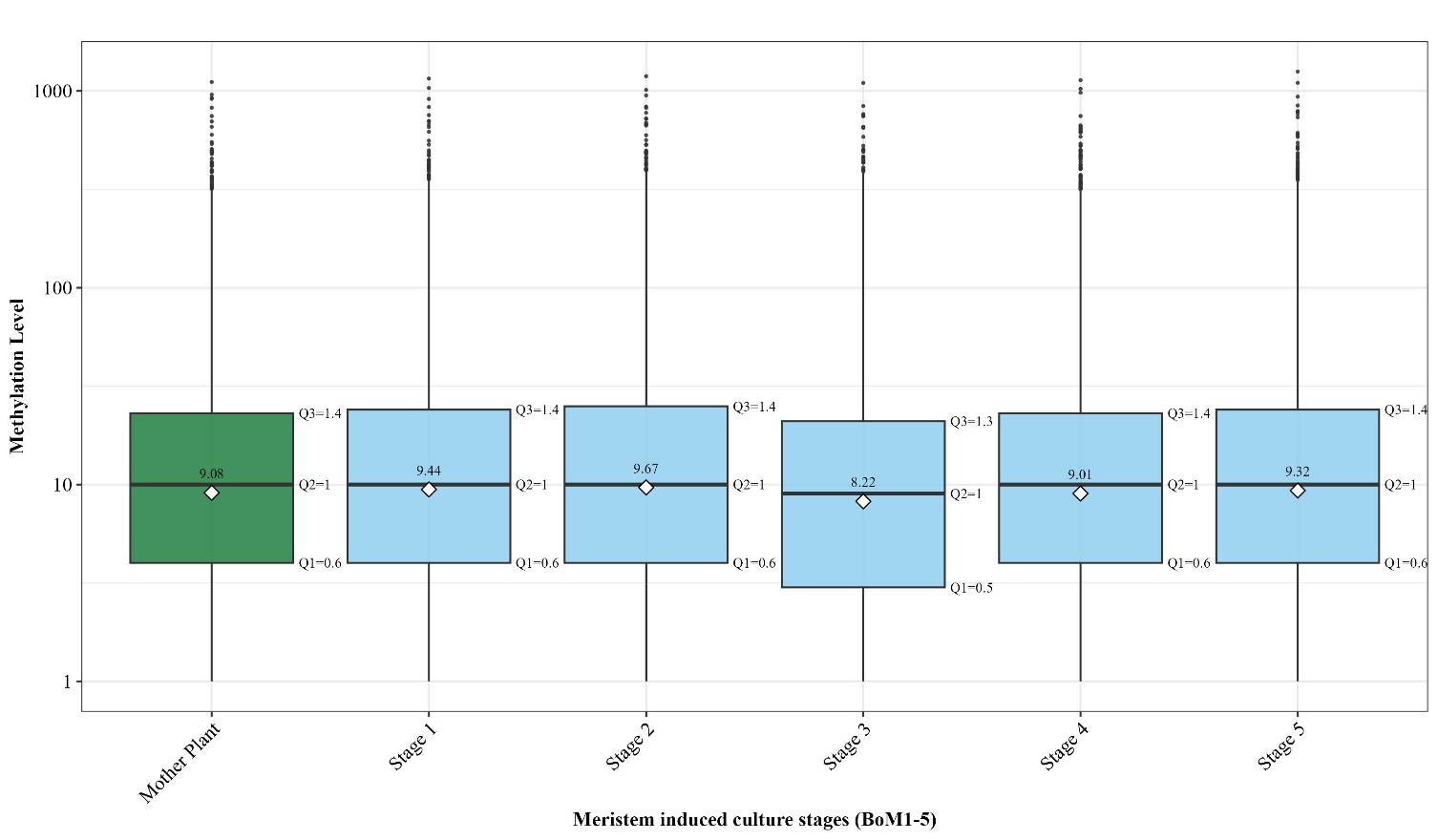


**D**
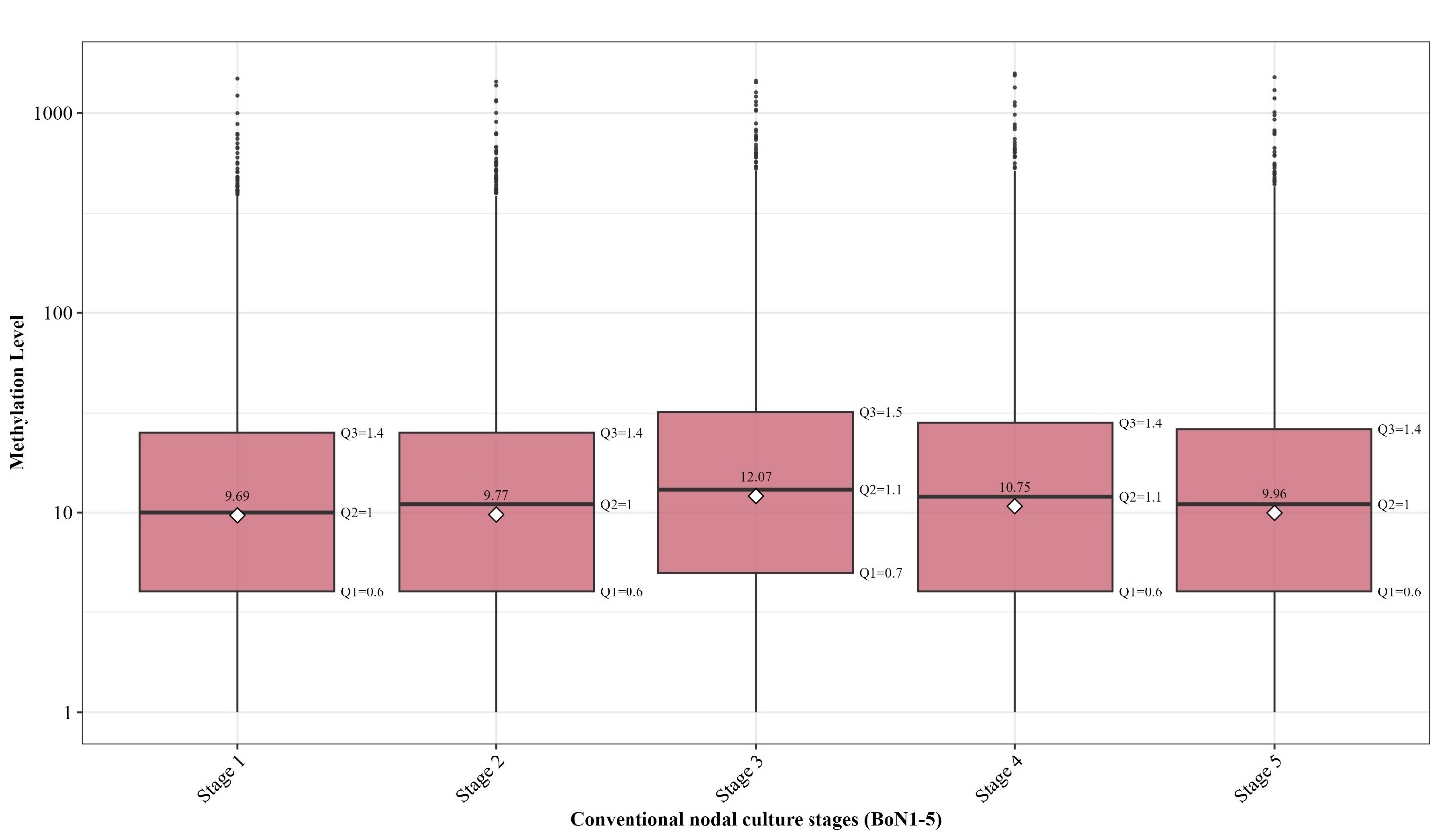


**E**
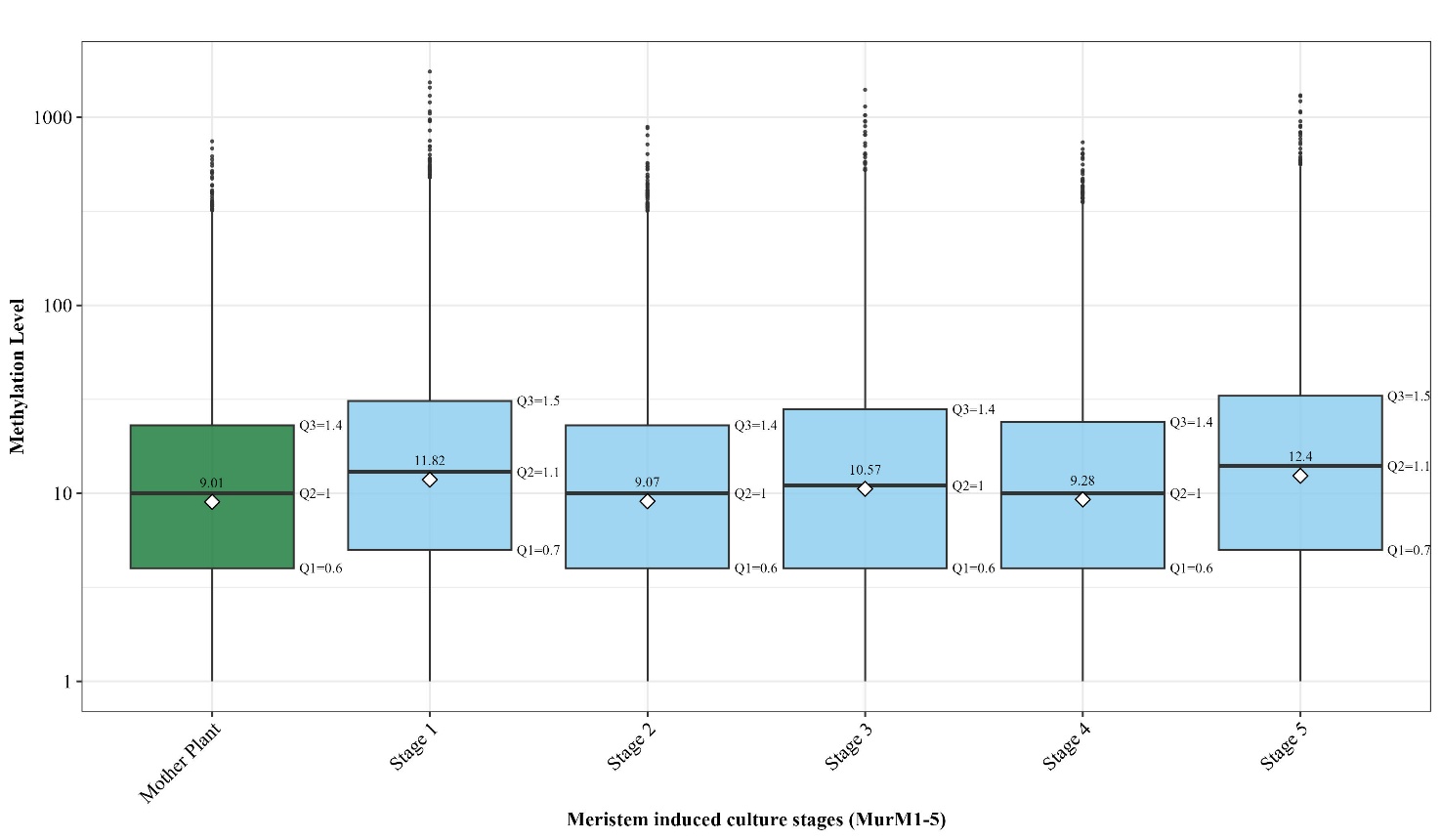


**F**
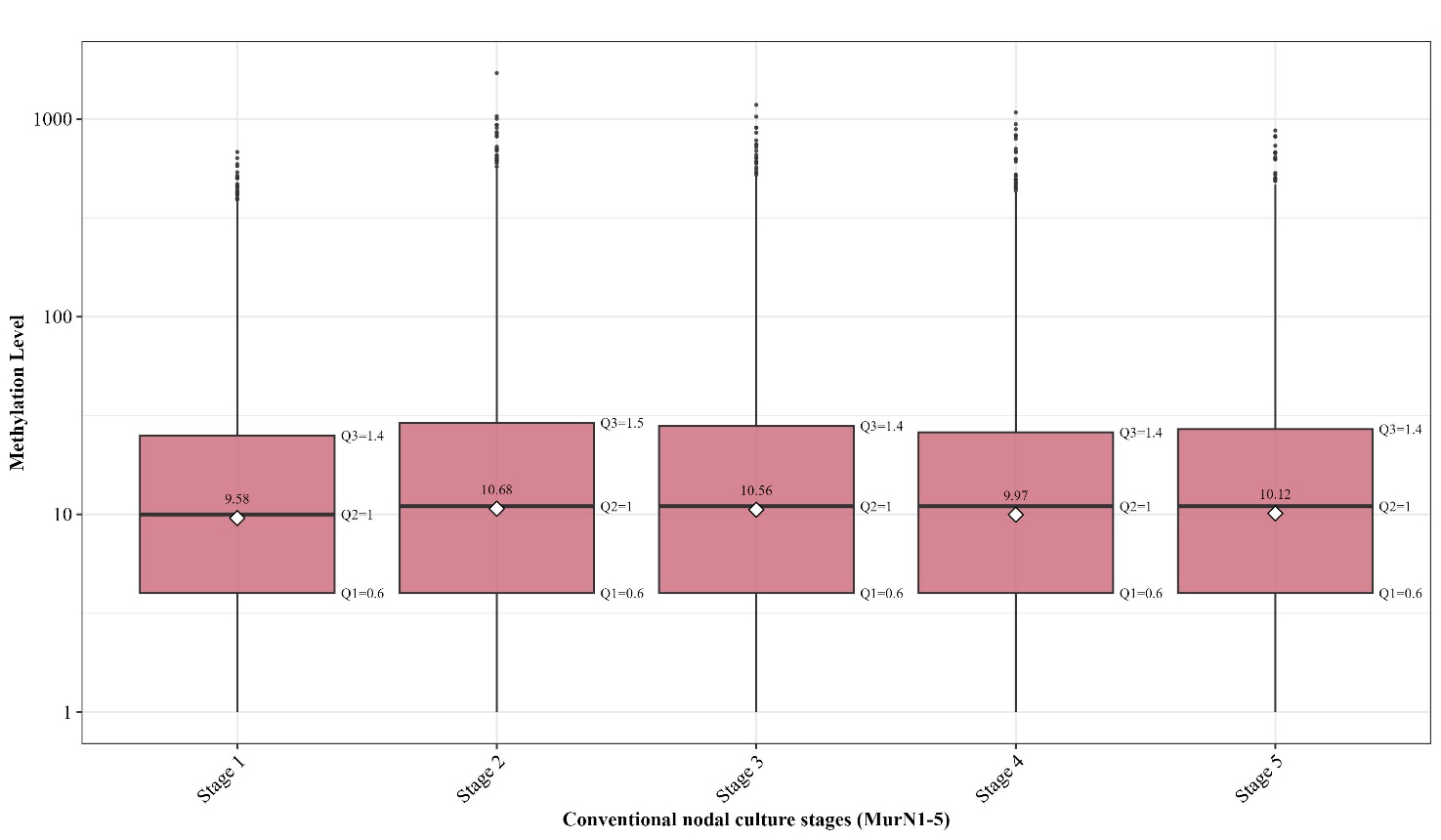


**Supplementary Figure S8** Chromosome-level analysis of exonic hypermethylation across tissue culture stage in half sibs of **(A)** Bellevue, **(B)** Bonita, and **(C)** Murasaki-29 sweetpotato varieties measured for methylation context (CG, CHG and CHH). Bars represent the mean methylation levels ± standard error (SE) for Meristem initiated nodal culture (M1–M5), conventional nodal culture (N1–N5), and the mother plant (MP). Different letters above bars denote significant differences between treatments (ANOVA followed by Fisher's LSD post-hoc test, p < 0.05). Vertical dashed lines separate the meristem, mother plant, and conventional nodal culture stages.

**A**
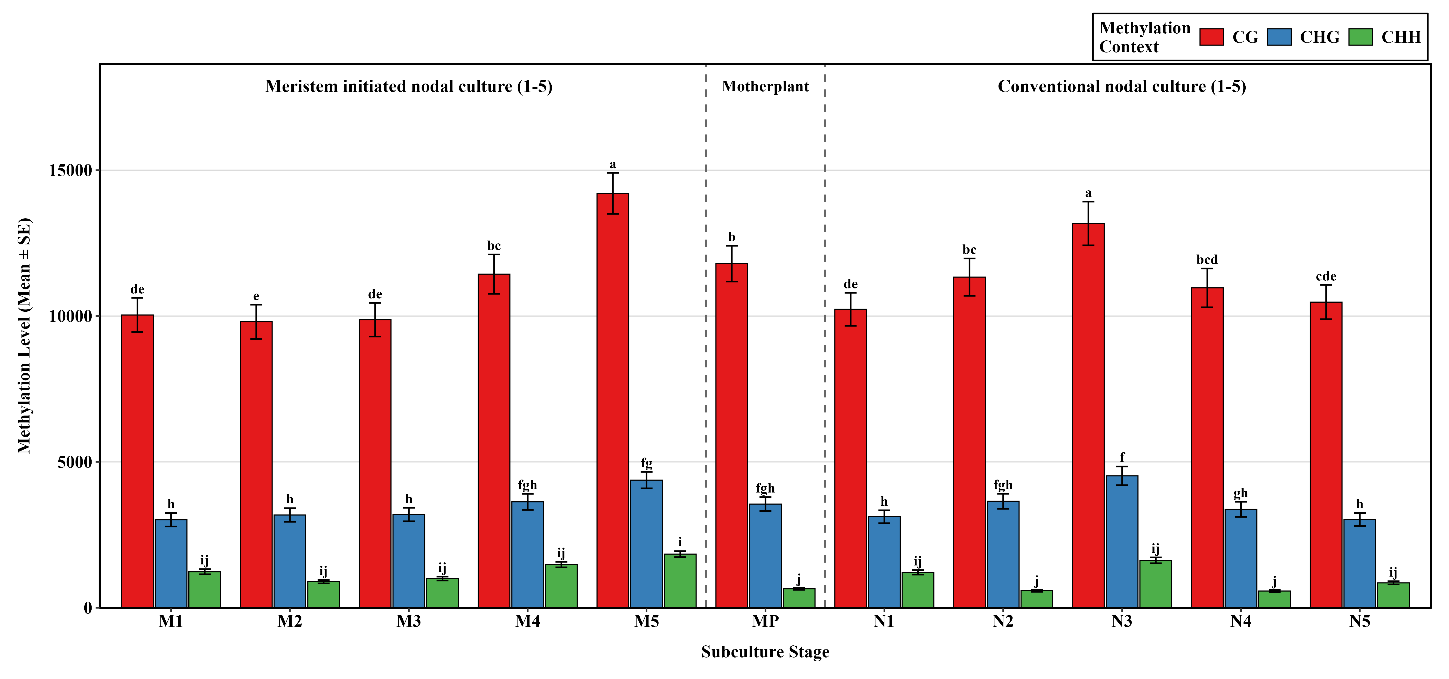


**B**


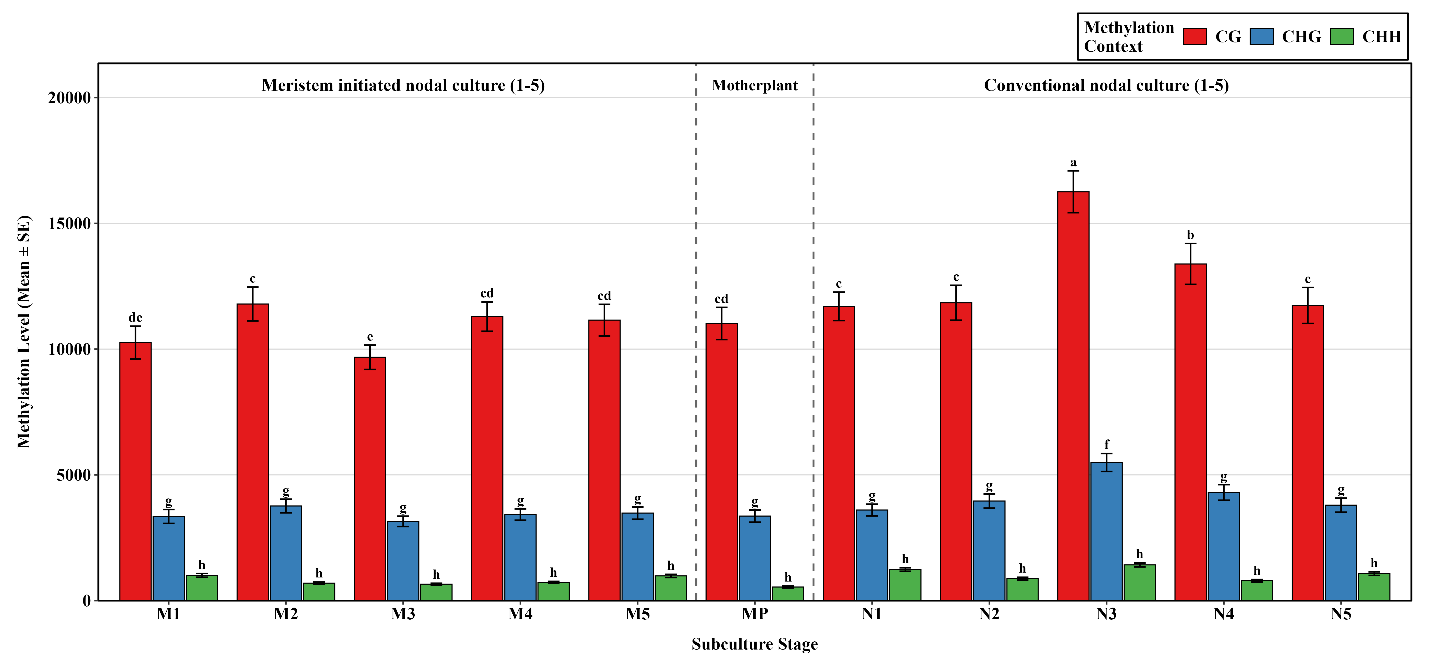


**C**


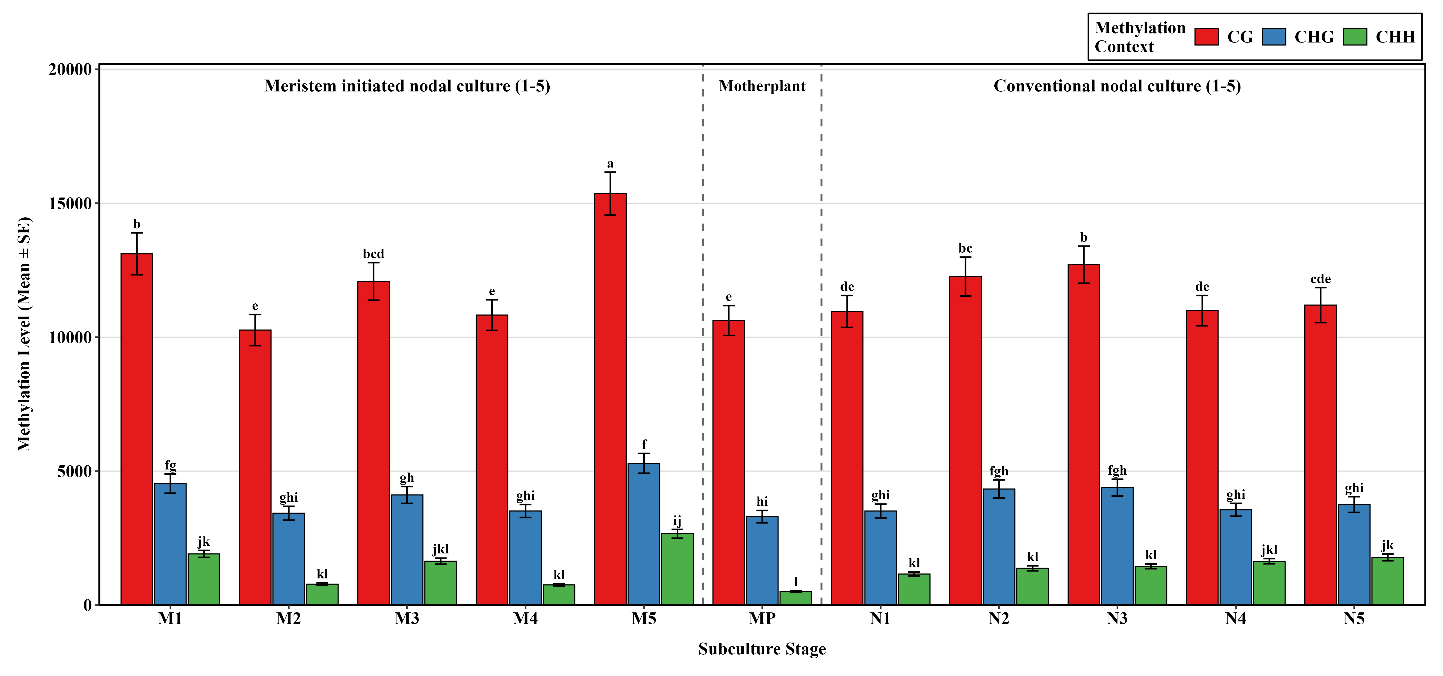


**Supplementary Figure S9** Variability across loci (gene-level) analysis across different drought treatment in three sweetpotato varieties **(A)** Bellevue, **(B)** Bonita and **(C)** Murasaki-29. Three different boxplot in different colors each representing different drought treatment are shown. Mean value is given with diamond shape label in center while quartiles are presented outside the boxes.

**A**


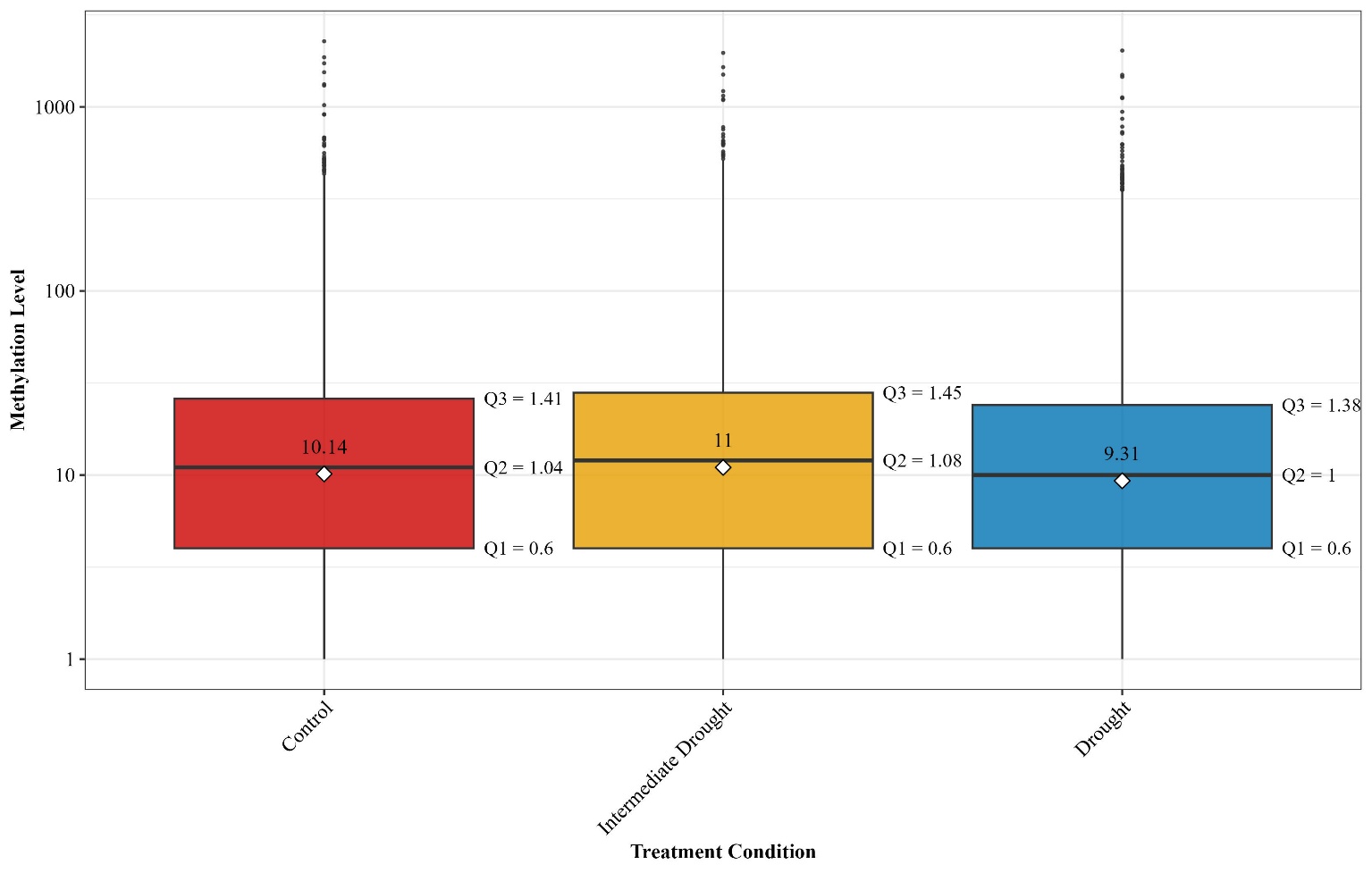


**B**


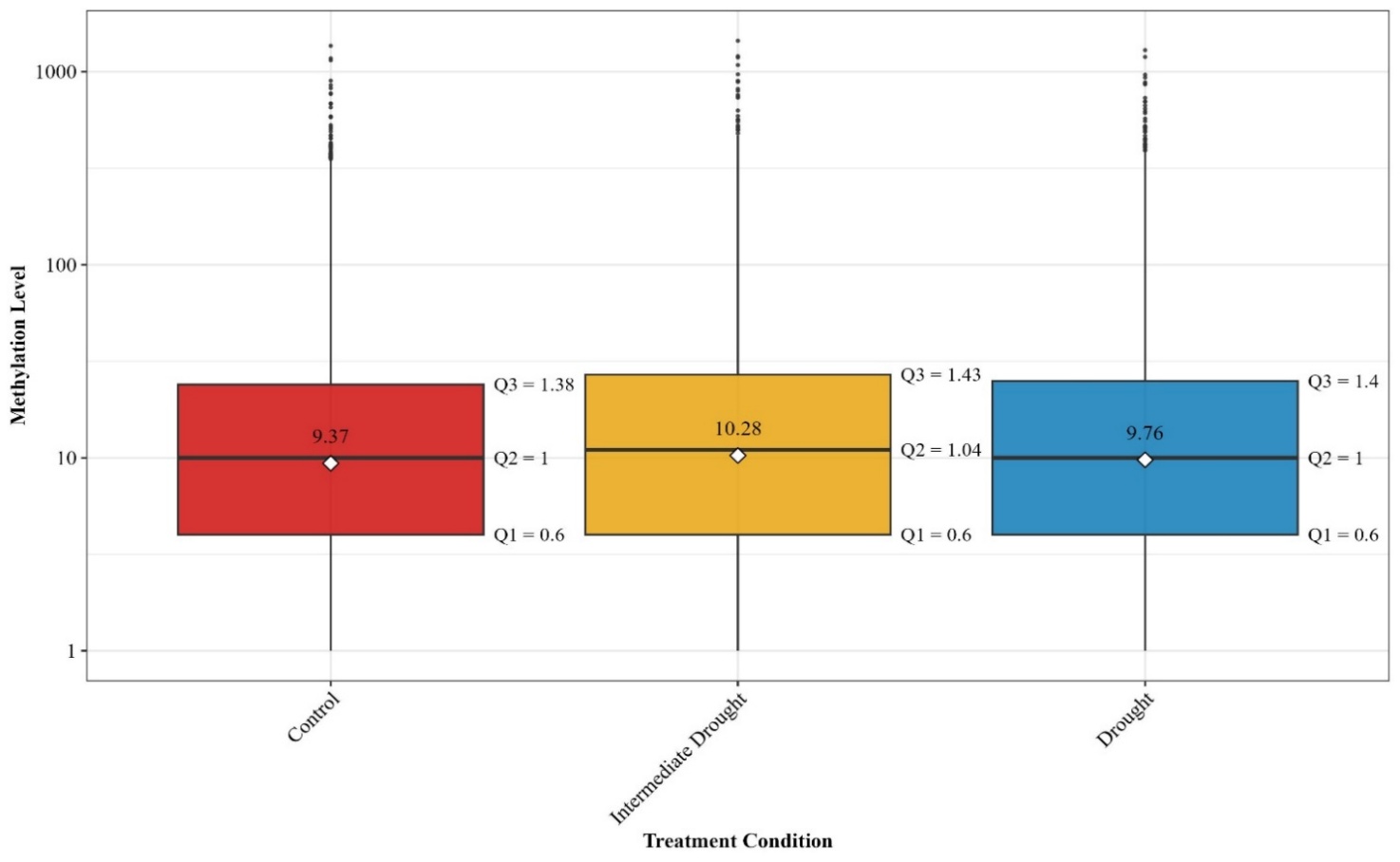


**C**


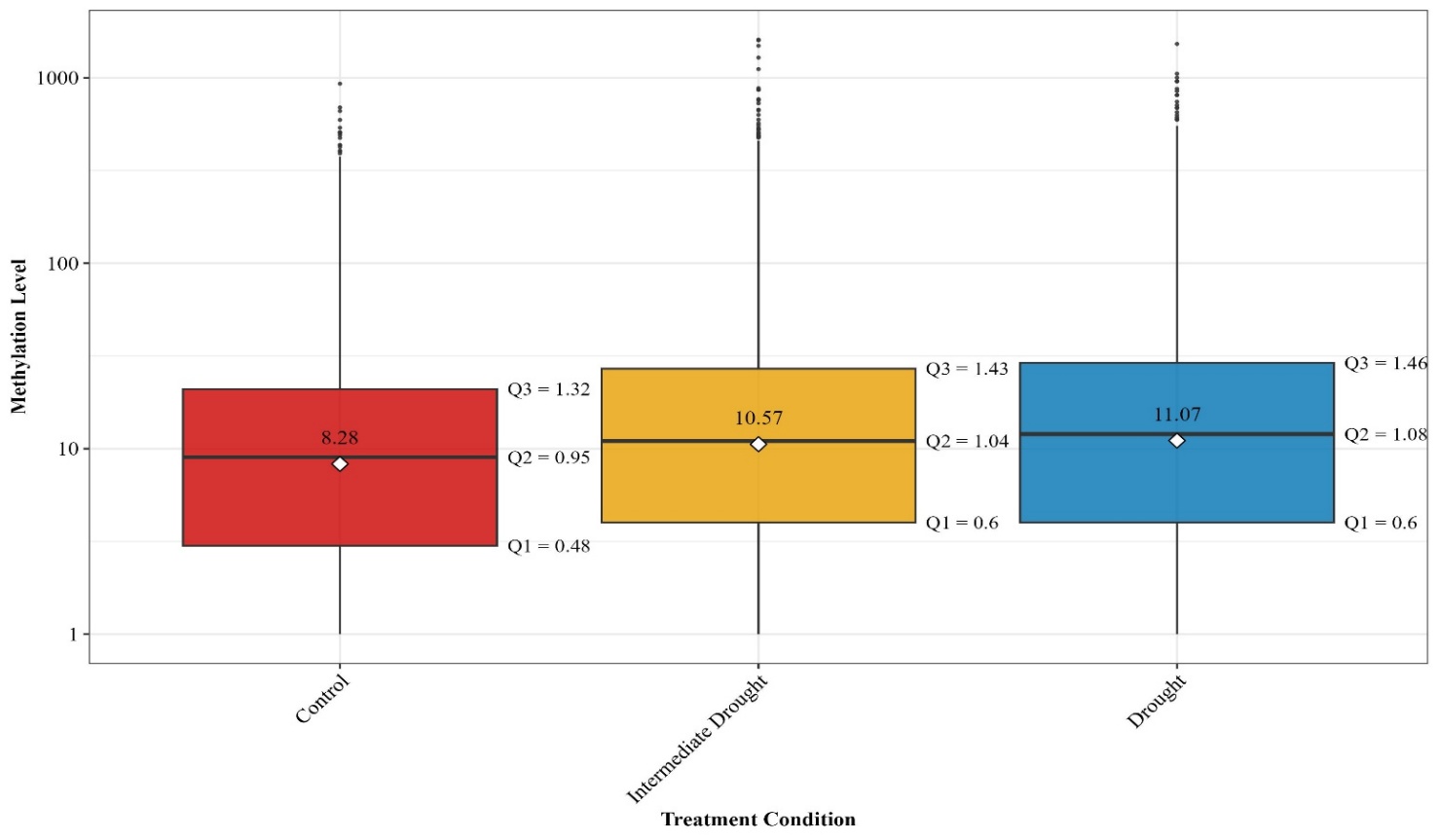


**Supplementary Figure S10** Chromosome-level analysis of exonic hypermethylation under drought stress in **(A)** Bellevue, **(B)** Bonita, and **(C)** Murasaki-29 sweetpotato varieties. Different letters above bars denote significant differences between treatments (ANOVA followed by Fisher's LSD post-hoc test, p < 0.05).

**A**


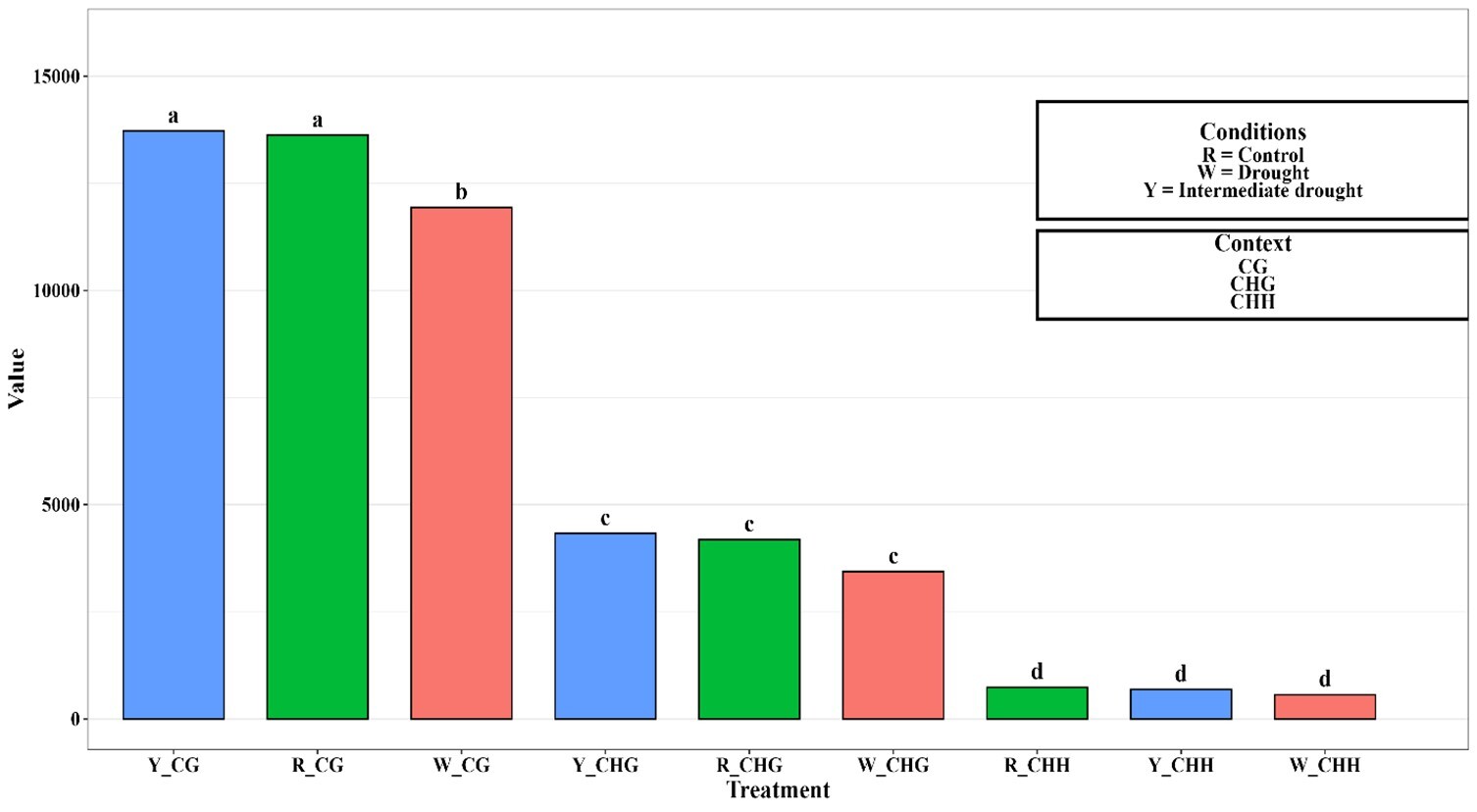


**B**


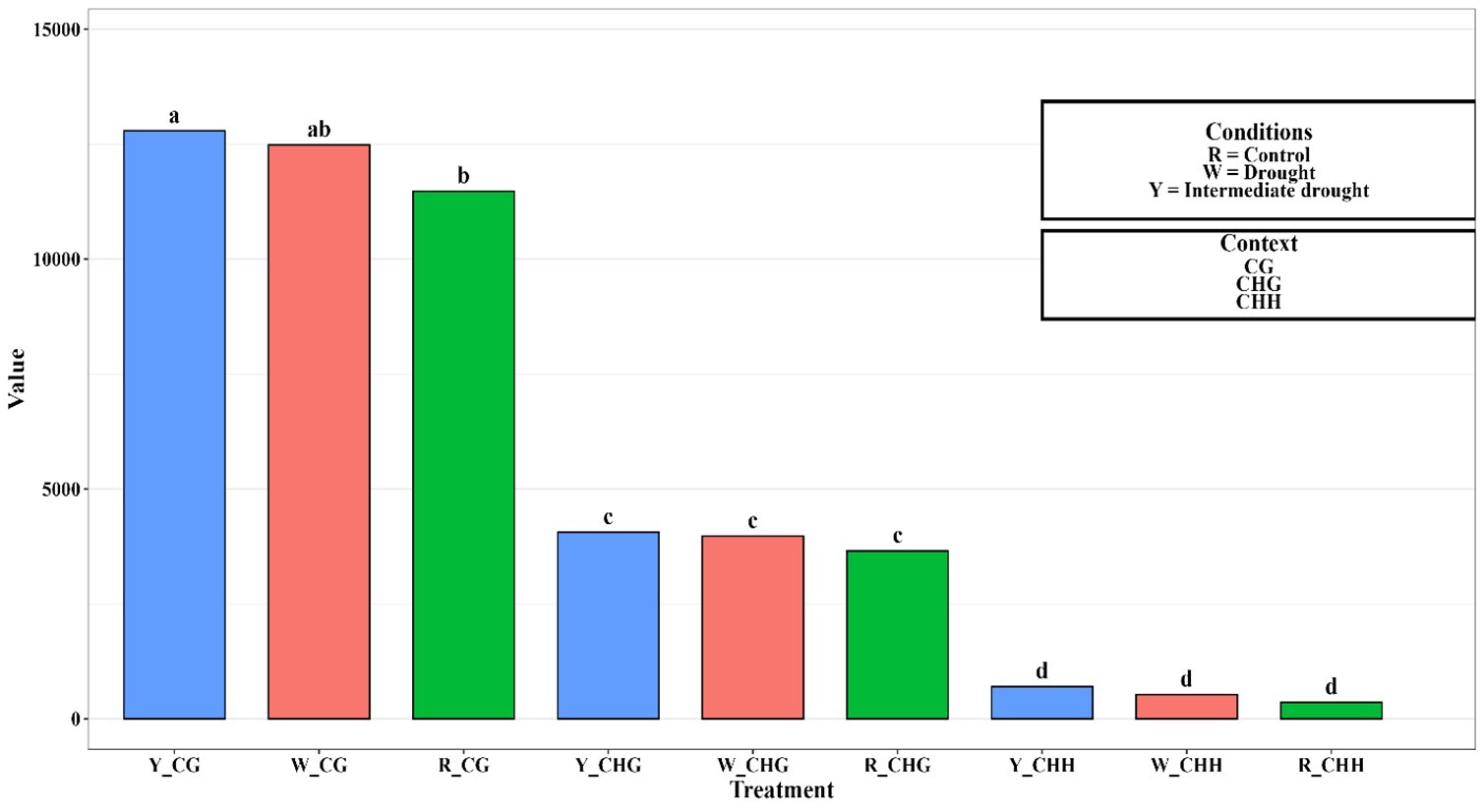


**C**


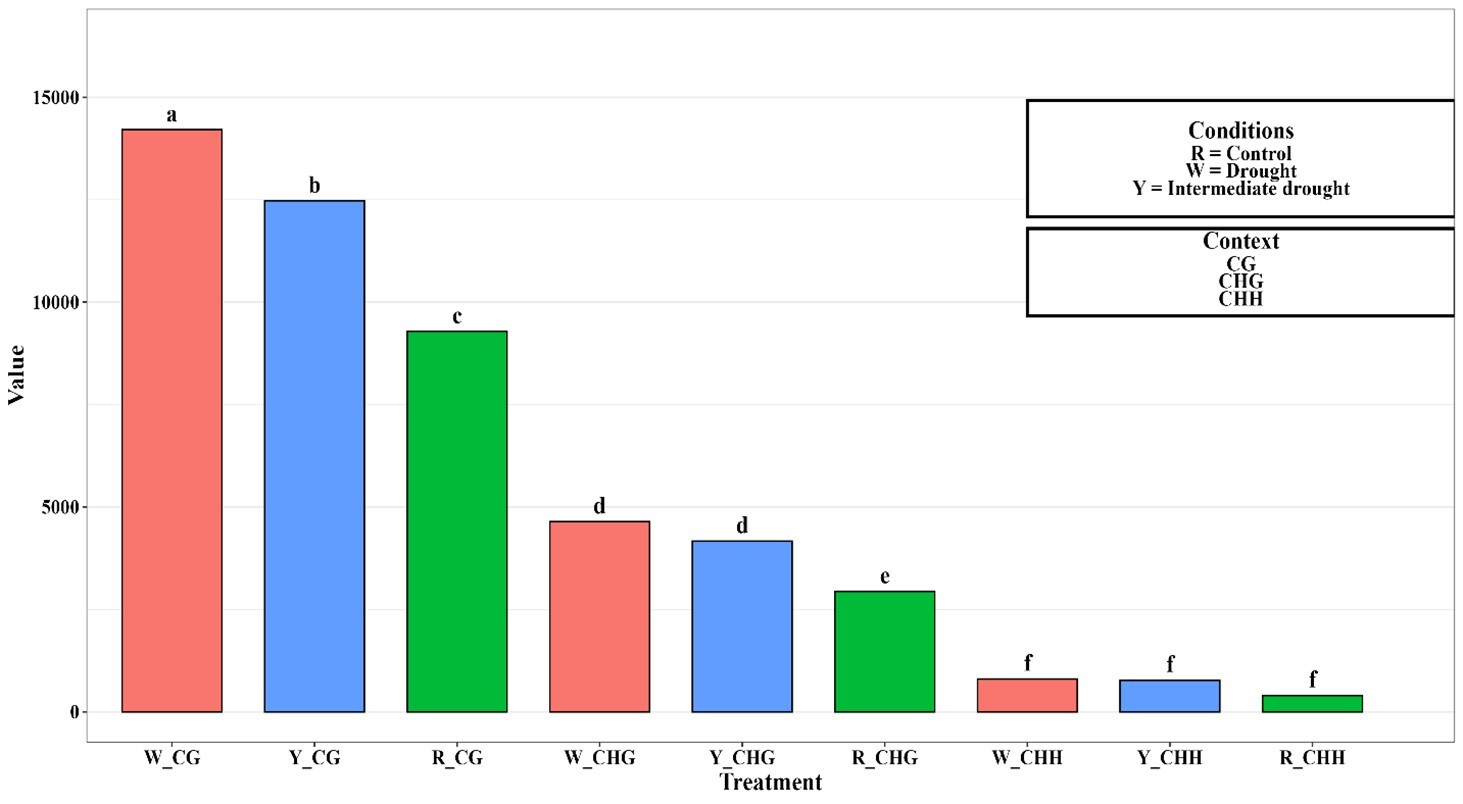

Supplement: Supplementary file 12 [file Table12.docx]
